# Topsentinol L Trisulfate, a Marine Natural Product That Targets Basal-like and Claudin-Low Breast Cancers

**DOI:** 10.3390/md19010041

**Published:** 2021-01-18

**Authors:** Nader N. El-Chaar, Thomas E. Smith, Gajendra Shrestha, Stephen R. Piccolo, Mary Kay Harper, Ryan M. Van Wagoner, Zhenyu Lu, Ashlee R. Venancio, Chris M. Ireland, Andrea H. Bild, Philip J. Moos

**Affiliations:** 1 Department of Pharmacology and Toxicology, University of Utah, Salt Lake, UT 84112, USA; nader.el.chaar@gmail.com (N.N.E.-C.); gajushrestha@gmail.com (G.S.); 2 Department of Medicinal Chemistry, University of Utah, Salt Lake, UT 84112, USA; tom.e.smith@utexas.edu (T.E.S.); MK.Harper@pharm.utah.edu (M.K.H.); ryan.vanwagoner@gmail.com (R.M.V.W.); Zhenyu.Lu@hsc.utah.edu (Z.L.); Ashlee.Venancio@utah.edu (A.R.V.); chris.ireland@utah.edu (C.M.I.); 3 The College of Life Sciences, Brigham Young University, Provo, UT 84606, USA; stephen.piccolo.byu@gmail.com; 4 Department of Medical Oncology and Therapeutics Research, City of Hope, Monrovia, CA 91016, USA

**Keywords:** topsentinol L trisulfate, AMPK, CHK1, breast cancer, drug susceptibility

## Abstract

Patients diagnosed with basal-like breast cancer suffer from poor prognosis and limited treatment options. There is an urgent need to identify new targets that can benefit patients with basal-like and claudin-low (BL-CL) breast cancers. We screened fractions from our Marine Invertebrate Compound Library (MICL) to identify compounds that specifically target BL-CL breast cancers. We identified a previously unreported trisulfated sterol, i.e., topsentinol L trisulfate (TLT), which exhibited increased efficacy against BL-CL breast cancers relative to luminal/HER2+ breast cancer. Biochemical investigation of the effects of TLT on BL-CL cell lines revealed its ability to inhibit activation of AMP-activated protein kinase (AMPK) and checkpoint kinase 1 (CHK1) and to promote activation of p38. The importance of targeting AMPK and CHK1 in BL-CL cell lines was validated by treating a panel of breast cancer cell lines with known small molecule inhibitors of AMPK (dorsomorphin) and CHK1 (Ly2603618) and recording the increased effectiveness against BL-CL breast cancers as compared with luminal/HER2+ breast cancer. Finally, we generated a drug response gene-expression signature and projected it against a human tumor panel of 12 different cancer types to identify other cancer types sensitive to the compound. The TLT sensitivity gene-expression signature identified breast and bladder cancer as the most sensitive to TLT, while glioblastoma multiforme was the least sensitive.

## 1. Introduction

Gene-expression profiling has identified five molecular subtypes of breast cancer, known as luminal A, luminal B, human epidermal growth factor receptor 2 (HER2)-enriched, claudin-low, and basal-like, with inter-subtype differences in incidence, survival, and treatment response [1,2,3,4,5]. With the molecular evaluation of thousands of breast cancer samples from The Cancer Genome Atlas (https://cancergenome.nih.gov), the International Cancer Genome Consortium (https://icgc.org), and the Molecular Taxonomy of Breast Cancer International Consortium (https://www.cbioportal.org), the stratification of breast cancer samples can be identified by combining DNA copy number alterations with gene expression data [6]. This stratification still identified basal-like breast cancer as a unique subtype. Among patients, breast cancer patients diagnosed with the basal-like molecular subtype exhibit a particularly poor prognosis and suffer from limited treatment options [7]. Basal-like breast cancer represents 10–25% of all breast carcinomas, generally occurring at an early age (<40 years old), with higher frequency in women of African origin [8]. Approximately 50–70% of all basal-like cancer patients lack the expression of estrogen receptor (ER), progesterone receptor (PR), and HER2, and therefore are clinically described as being triple negative. Basal-like breast cancer manifests as a highly aggressive tumor that is responsive to chemotherapy [7]. However, patient prognosis remains poor with basal-like cancer exhibiting a high recurrence rate and low patient survival [2]. At the molecular level, basal-like breast cancer exhibits expression patterns which are also observed in the basal epithelial layer of the skin and airways; this includes expression of high molecular weight cytokeratins 5, 6, and 17 and deficiencies in retinoblastoma transcriptional corepressor 1 (RB1), BRCA1, and tumor protein 53 (TP53). Moreover, a high rate of aneuploidy is observed in these tumors, reflective of increased genetic instability [7,8].

Interestingly, the claudin-low subtype shares some similarities in gene-expression features with the basal-like subtype such as low expression of HER2 and the luminal gene clusters, indicating genomic similarities between the two subtypes [9]. Moreover, similar to the basal-like subtype, claudin-low tumors are also triple negative but the prognosis is similar to many of the intrinsic categories [10], including a basal-like claudin-low category with a poorer prognosis. However, claudin-low breast cancers remains as an individual subtype, characterized by the minimal expression of several claudin genes, such as claudin 3, 4, and 7, which are involved in epithelial cell tight-tight junctions. Basal-like and claudin-low (BL-CL) tumors also lack cell-cell junction proteins, such as E-cadherin, and almost always are characterized by having an intense immune cell infiltrate, stem cell properties, and features of epithelial-mesenchymal transition [3,4,8,11,12]. Due to the non-luminal molecular nature of these two subtypes, and the lack of known protein targets on these cancers, few effective treatment options are available. As such, there is an urgent need to identify new therapeutic leads and potential targets that can improve patient prognosis.

The Marine Invertebrate Compound Library (MICL) is a unique resource that serves as a platform for discovery of novel small molecule-mediated biological activities in a variety of systems. The MICL is derived from an extensive collection of small-molecule natural products isolated from over 1200 unique marine organisms (85% sponges from over 150 genera, 12% tunicates, and 3% other phyla) collected from diverse locations around the world over the past twenty years [13,14]. Natural products tend to be more complementary in shape to their targets [15], due to their development in a competitive ecological selection process that favors the production of compounds with strong biological activity [16,17,18]. Around 75% of all anticancer drugs developed between 1940 and 2014 were either derived from or inspired by natural products [19]. In particular, several marine natural products have been shown to exhibit anticancer properties, such as didemnin B, aplidine, and ecteinascidin-743, the latter of which succeeded in passing clinical trials in Europe and was approved by the European Commission for the treatment of refractory soft-tissue sarcomas in 2007 [20]. As of 2016, there were seven drugs approved by the United States Food and Drug Administration that are derived from marine natural products, four of which target cancers, and 15 more marine natural products in clinical trials, of which 12 are anticancer [21]. In summary, marine natural products have proven their potential for development into clinically useful drugs [20,21].

In this study, we performed a stepwise screening approach with 2778 fractions from the MICL and identified a previously uncharacterized trisulfated sterol, topsentinol L trisulfate (TLT), purified from a marine sponge *Topsentia* sp. (PNG07-3-073) collected from Papua New Guinea (Supplemental Figure S1). TLT, as well as its parent fraction and subfraction, exhibited increased effectiveness against basal-like and claudin-low BL-CL cell lines as compared with luminal and HER2+ subtypes. We showed that treating BL-CL cell lines with TLT led to the inhibition of AMPK and CHK1, using biochemical and proteomic analyses. To validate the importance of inhibiting the activity of AMPK/CHK1 in BL-CL cell lines, we tested breast cancer cell lines with known small molecule inhibitors of AMPK and CHK1, i.e., dorsomorphin and Ly2603618, respectively, and observed that they were significantly more effective against BL-CL cell lines than luminal and HER2+ subtypes. Furthermore, overexpressing AMPK or CHK1 in BL-CL cell lines sensitized them to TLT treatment. We also generated a genomic gene-expression signature of TLT sensitivity and projected it against a panel of human patient tumors of twelve different cancer types, which identified breast and bladder cancer as the two cancers most sensitive to TLT. Overall, this study incorporates the genomic classification of breast cancer to high-throughput drug screening and identifies a novel small molecule, TLT, selective against BL-CL cell lines. Therefore, the work described here sheds light on the importance of targeting AMPK or CHK1 in this molecular subtype and suggests the potential of these proteins as therapeutic targets in BL-CL cell lines.

## 2. Results

### 2.1. Identification of Topsentinol L Trisulfate as a Selective Inhibitor of Basal-Like and Claudin-Low BL-CL Breasst Cancers

**Figure 1:**
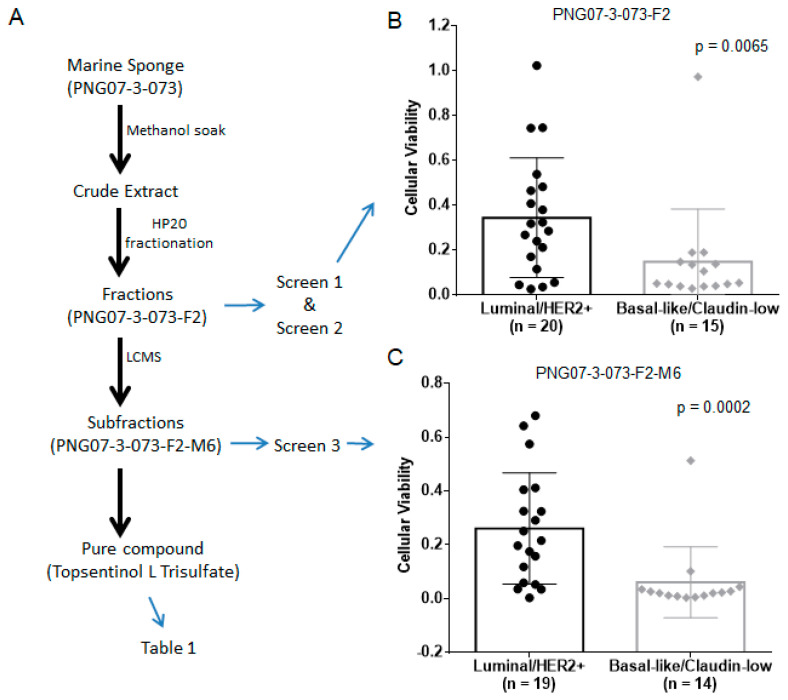
The overall design of the stepwise drug screen. (**A**) The path towards identifying topsentinol L trisulfate (TLT). Marine sponge PNG07-3-073 was diced and soaked in methanol to obtain a crude extract, which was fractionated on HP20SS resin. Among the five fractions obtained, only the F2 fraction exhibited tumoricidal activity (Screen 1); (**B**) The viability data for fraction PNG07-3-073-F2, this activity was observed against basal-like and claudin-low (BL-CL) cell lines (Screen 2). Then, PNG07-3-073-F2 was further fractionated, and the subfractions investigated for anti-BL-CL activity, where the M6 fraction was identified as being BL-CL selective (Screen 3); (**C**) The viability data for fraction PNG07-3-073-F2-M6. Large-scale isolation of PNG07-3-073 ensued, culminating in the purification of TLT and its identification as the active compound in PNG07-3-073 responsible for anti-BL-CL effects (Table 1). Cell lines were treated and the bar graphs (with standard deviation) with all data displayed as scatter dot diagrams of the viability assessment of each cell line.

The goal of this study was to isolate a novel inhibitor of BL-CL breast cancers, describe the pathways effectively blocked by the compound, and project treatment efficacy across other cancer types. We aimed at achieving this through a stepwise approach consisting of the following steps: (1) screening MICL for fractions that exhibited tumoricidal properties, (2) selecting candidate fractions that displayed effectiveness against BL-CL cell lines, (3) separating the active compounds in the fractions, (4) identifying the active compound with anti-BL-CL properties, (5) describing the cell signaling effects of the compound on BL-CL activity, (6) projecting TLT sensitivity across human tumors of various cancer types using a gene-expression signature (Figure 1A).

We used a bioassay-guided fractionation approach to identify potential compounds with tumoricidal activity, beginning by screening 2778 synthetic polymer resins for high-performance liquid chromatography (HP20) fractions from MICL against a panel of 16 cell lines. These fractions represent complex mixtures and served as a starting point to identify promising hits [13,14]. We selected 107 fractions based on differential tumoricidal activity, eliminating all fractions that were universally toxic or showed minimal anticancer activity. We added 85 previously unscreened HP20 fractions from MICL based on chemical similarity to the 107 selected fractions guided by chemo-taxonomic considerations of source organisms [13,14]. The combined 192 fractions were screened against a panel of 35 breast cancer cell lines and 37 lung cell lines to identify fractions more effective against breast cancer, and in particular against BL-CL breast cancers (Figure 1A Screen 2). Among these, 34 fractions were selective for breast cancer, of which seven displayed subtype selectivity against BL-CL activity (Figure 1B, Screen 2). These seven fractions were further fractionated by LCMS into 20 subfractions (M1–M20) each and screened at two doses against a panel of 33 breast cancer cell lines (Figure 1C, Screen 3). This screen identified five subfractions with significant selectivity against BL-CL cell lines, out of which three subfractions originated from the marine sponge PNG07-3-073 F2 fraction. We analyzed the results of all three screens retrospectively and observed that PNG07-3-073–F2 was initially identified as a candidate with increased tumoricidal activity against BL-CL breast cancer (Figure 1B). The HP20 fractionation of the sponge also resulted in 4 other fractions, i.e., FW, F1, F3, and F4; however, none exhibited sufficient tumoricidal activity, and therefore did not proceed past the first screen. The F2 was the only fraction from the *Topsentia* sponge PNG07-3-073 to exhibit anti-BL-CL activity (Figure 1B). This activity was maintained after further fractionation of F2 via LCMS into 20 subfractions. Among the 20 subfractions, in particular, the M6 subfraction displayed significant effectiveness against BL-CL activity (Figure 1C). From these screening experiments, BL-CL cell lines exhibit significantly lower cell viability when treated with PNG07-3-073-F2 and PNG07-3-073-F2-M6 than luminal/HER2+ cell lines.

**Figure 2:**
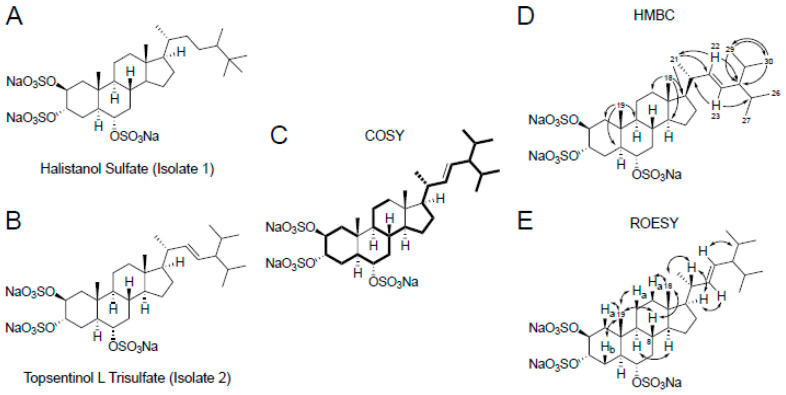
Structure elucidation of topsentinol L trisulfate, isolated from the marine sponge *Topsentia* sp. (PNG07-3-073), by two-dimensional (2D) nuclear magnetic resonance (NMR). Chemical structures of (**A**) halistanol sulfate and (**B**) topsentinol L trisulfate. The chemical structure of TLT was deciphered from (**C**) homonuclear correlation spectroscopy (COSY) correlations between protons of immediately adjacent carbon atoms (shown in bold); (**D**) Heteronuclear multiple bond correlation (HMBC) spectroscopy (unidirectional arrows represent correlations between methyl or alkene protons and carbon atoms 3 bonds away); (**E**) Through-space proton-proton rotating frame Overhause effect spectroscopy (ROESY) correlations (bidirectional arrows) that reveal the configurations of key stereocenters.

Our next step was to proceed with the identification of the active compounds in the M6 subfraction to isolate any compounds inducing this response. We performed a scaled-up extraction of bulk PNG07-3-073 sponge and purified two compounds, i.e., isolate 1 and 2. One-dimensional (1D) nuclear magnetic resonance (NMR) analysis (Table S3) identified isolate 1 as the previously reported metabolite halistanol sulfate [22] (Figure 2C and Figure S2A,B) and this was validated by using low-resolution mass spectroscopy (Figure S3A). Isolate 2 was identified via 1D (Figure S2C,D) and two-dimensional (2D) (Figure S4) NMR analysis (Table S3) as a previously uncharacterized sulfated sterol identical to the known compound topsentinol L but with three sulfate groups [23] (Figure 2). The structure of the compound was corroborated by the data obtained through low-resolution mass spectroscopy (Figure S3B). We named this compound topsentinol L trisulfate, or TLT (Figure 2). Both structures were validated by high-resolution electrospray ionization mass spectrometry (Supplementary Methods). Then, halistanol sulfate and TLT were screened against a panel of 30 breast cancer cell lines and tested for effectiveness against BL-CL activity (Table 1). TLT showed greater tumoricidal activity against BL-CL breast cancers in 8 of 12 breast cancer cell lines (50% effective concentration (EC50) <100 µM) as compared with luminal/Her2+ breast cancer, for which TLT demonstrated similar potency in only 4 of 18 cell lines (Table 1, Figure S5). Halistanol sulfate demonstrated considerable cytotoxicity but did not exhibit differential activity between BL-CL and luminal/Her2+ cell lines (*p* = 0.247, Figure S5). Through a multi-step screening and fractionation process, we identified a novel sulfated sterol, TLT, which exhibited significant subtype selectivity against BL-CL activity.

### 2.2. Topsentinol L Trisulfate Treatment Inhibits AMPKα and CHK1 but Activates p38

**Figure 3:**
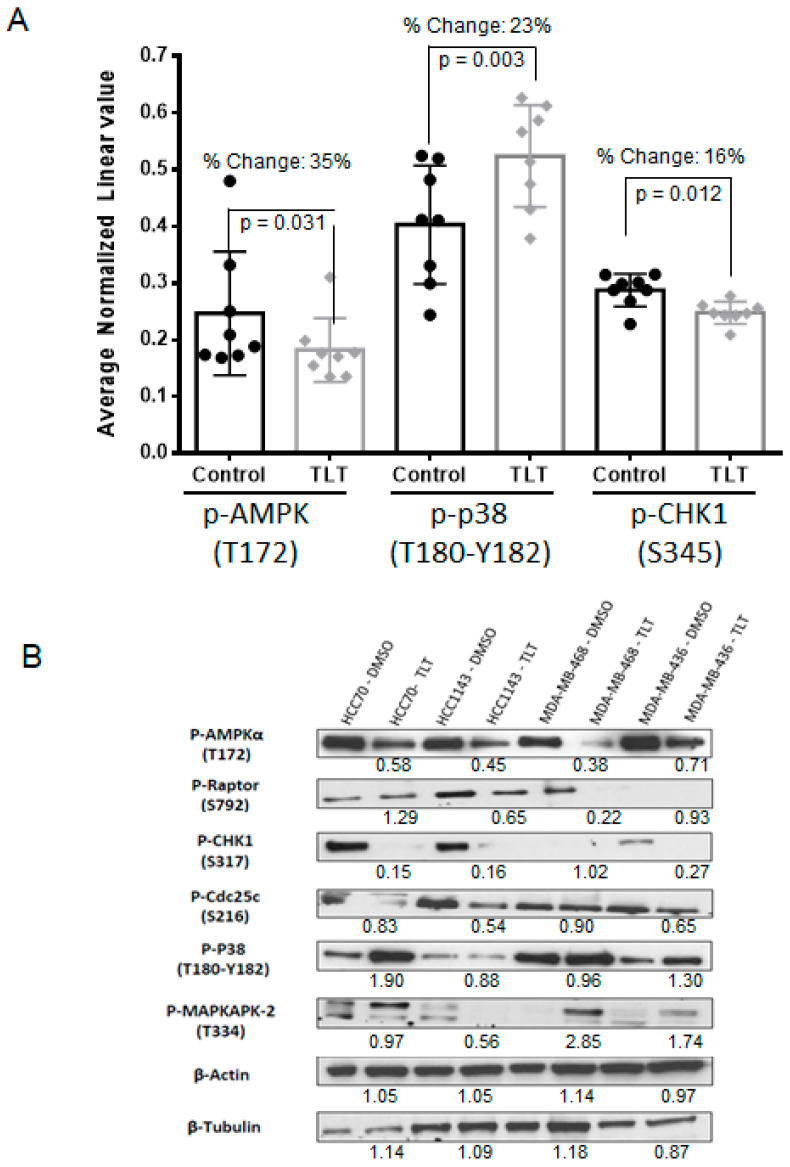
TLT inhibits AMPK and CHK1 but activates p38. (**A**) A panel of 8 BL-CL cell lines were treated with TLT at a 105 µM dose (approximate average EC75 dose across all 8 lines) for 6 h and compared with the DMSO control via reverse-phase protein array (RPPA) that investigated 217 proteins. Eight proteins displayed 15% or more significant deregulation in protein levels, among which, AMPK, CHK1, and p38 are displayed here in bar graphs (with standard deviation) with all data displayed as scatter dot diagrams; (**B**) Observations made for p38, AMPK, and CHK1 in the RPPA experiment were validated by Western blotting. A panel of four BL-CL cell lines were treated similarly with TLT at a 105 µM dose for 6 h and compared to the DMSO control. Beta-actin and β-tubulin were used here as protein loading controls. The values under the TLT treatments are the relative densitometry as compared with the DMSO vehicle treatment for each cell line.

Our next goal was to analyze and describe the signaling effects that are induced by TLT in cancer cells. For this purpose, we treated eight TLT-sensitive BL-CL cell lines with TLT or DMSO control and screened for changes in the phosphorylation of 217 proteins using a reverse-phase protein array (RPPA) [24]. From this screen, we identified 21 proteins that exhibited a 15% or greater change in phosphorylation level, with only eight of these proteins exhibiting statistically significant changes (Supplementary Table S4). To focus our research and narrow down our investigation, we selected three proteins, i.e., AMPK, CHK1, and p38, based on fold change and statistical significance. Note that the non-phosphorylated forms of these proteins demonstrated negligible differences in protein levels (Supplementary Table S4), and therefore we focused on the phosphorylated forms. AMPK phosphorylation recorded the largest statistically significant change as compared with the DMSO control among all proteins, with a 35% reduction in Thr172 phosphorylation, required for AMPK activation [25] (*p* = 0.031, Figure 3A and Supplementary Table S4). Validating the reduction in AMPK phosphorylation, phosphorylation of ACC, a direct downstream effector of AMPK [26], was also significantly reduced (23.6% reduction in Ser79, *p* = 0.024, Table S4). We also observed significant changes in phosphorylation of CHK1, recording a 16% downregulation in the nuclear localization mark Ser345 [27] (*p* = 0.012, Figure 3A and Table S4). CHK1 has recently been suggested to represent a therapeutic target in triple-negative breast cancer (TNBC) [28], and its reduced phosphorylation following TLT treatment could explain the selectivity of TLT against BC-CL breast cancers. Increases in p38 phosphorylation were among the significant changes observed upon treatment with TLT, with a 23% increase in Thr180-Tyr182 phosphorylation (*p* = 0.003, Figure 3A and Table S4). The activation of p38 could be particularly important as phosphorylation of p38 has been shown in other experiments to lead to the induction of apoptosis [29].

In order to validate the observed effects on AMPK, p38, and CHK1 phosphorylation following TLT treatment in the RPPA screen, we analyzed phosphorylation levels by Western blotting. Downregulation of AMPK phosphorylation was most consistent across all four cell lines (Figure 3B). Reduced CHK1 phosphorylation at the active site residue Ser317 [30] and increased p38 phosphorylation were also readily observed in two of the four cell lines (Figure 3B), confirming RPPA results (Figure 3A). In support of these changes in phosphorylation affecting enzyme function, three kinases, i.e., Raptor, Cdc25c, and MAPKAPK-2, representing downstream substrates of AMPK, CHK1, and p38, respectively [28,29,31], also showed changes in phosphorylation in at least two of the cell lines tested following TLT treatment (Figure 3B). These data describe the landscape of proteomic changes induced following TLT treatment in BL-CL cell

### 2.3. Inhibition of AMPK and CHK1, Alone or in Combination, is Effective against BL-CL Breast Cancers

**Figure 4:**
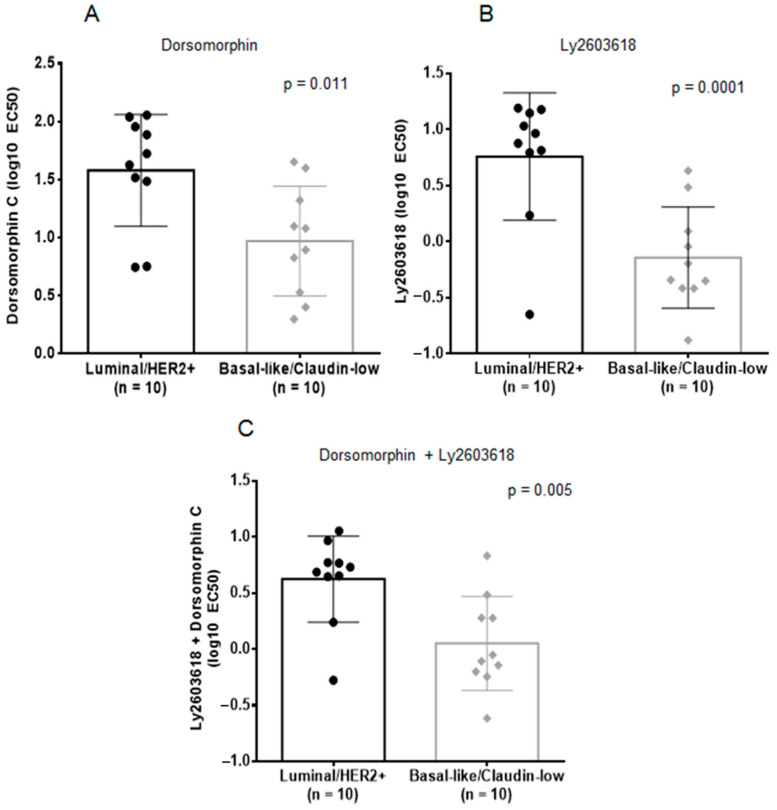
Single or dual inhibition of AMPK and CHK1 is selective against BL-CL cell lines. Cell lines were treated and the bar graphs (with standard deviation) with all data displayed as scatter dot diagrams of treatment EC50 values were plotted. Every dot represents the EC50 for a cell line. Luminal/HER2+ and BL-CL cell lines were treated with (**A**) the AMPK inhibitor, dorsomorphin, (**B**) the CHK1 inhibitor, Ly2603618, or (**C**) the concurrent treatment with dorsomorphin and Ly2603618 for 72 h. The differences were assessed using an un-paired *t*-test (*p* < 0.05 was considered significant).

Due to the consistency with which AMPK (either the protein itself or its downstream effector Raptor) and CHK1 (at two separate residues, Ser345 and Ser317) phosphorylation are affected, as well as the recent discovery of CHK1 treatment efficacy against TNBC [28], we decided to test the relevance of inhibiting AMPK and CHK1 in BL-CL cells. We investigated the effects of AMPK, CHK1, and AMPK1 + CHK1 small molecule targeted inhibition on a panel of twenty breast cancer cell lines. We screened the panel with dorsomorphin (an AMPK inhibitor [32]), Ly2603618 (a CHK1 inhibitor that has been used in clinical trials [33]), and the combination of both. Our results showed that either treatment strategy is significantly more potent against the BL-CL subtypes than luminal/HER2+ breast cancer (Figure 4). Dorsomorphin, on the one hand, was more than four times more potent against BL-CL breast cancers (average EC50 = 9.33 µM) as compared with luminal/HER2+ breast cancer (average EC50 = 37.87 µM), which was a significant difference (*p* = 0.011, Figure 4A). Ly2603618, on the other hand, was almost eight times more toxic against BL-CL breast cancers (average EC50 = 0.72 µM) as compared with luminal/HER2 breast cancer (average EC50 = 5.73 µM), which was also a significant difference (*p* = 0.001, Figure 4B). Interestingly, the combination therapy was also significantly more potent against BL-CL breast cancers (*p* = 0.005) (Figure 4C). To confirm that AMPK and CHK1 are part of the killing mechanism, we overexpressed AMPK and/or CHK1 in two BL-CL cell lines and performed dose-response assays with TLT to study the effect of AMPK and CHK1 abundance on drug response. We observed that the overexpression of AMPK and CHK1, or both concurrently, led to the sensitization of the cell lines to TLT treatment (Figure S6). Indeed, an increase in the availability of AMPK or CHK1 lowered the EC50 of TLT across both cell lines (Table S5), further linking the TLT killing mechanism to the AMPK and CHK1 proteins. These results demonstrate the importance of the inhibitory effects of TLT on AMPK and CHK1 in BL-CL cell lines and suggest the potential use of AMPK or CHK1 inhibition as a treatment against BC-CL breast cancers.

### 2.4. Topsentinol L Trisulfate (TLT) Sensitivity Signature Predicts Breast and Bladder Cancer Response in Human Tumors

**Figure 5:**
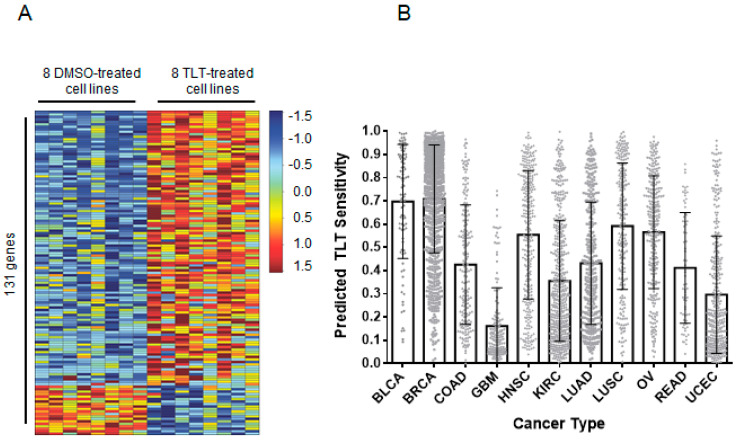
TLT sensitivity is predicted in breast and bladder cancers using gene-expression signature analysis. (**A**) The gene-expression signature for TLT sensitivity was generated by treating eight BL-CL cell lines with either TLT or DMSO. The heatmap columns are the eight cell lines making up eight controls on the left (treated with DMSO) and eight treated samples on the right (treated with TLT). Each row represents a gene that is part of the signature. There are a total of 131 genes making up the signature. Red indicates upregulation while blue indicates downregulation of the gene. (**B**) The TLT sensitivity signature was used to project the sensitivity of twelve different cancers. The results are shown in a bar graph where the x-axis represents the twelve cancer types assayed and the y-axis represents the predicted score of TLT sensitivity (minimum = 0 and maximum = 1). Each column portrays the mean of the TLT sensitivity scores across the samples in a particular cancer type. The error bars indicate the standard deviation. Breast and bladder cancers were predicted to be the most sensitive to TLT treatment, with glioblastoma being the least sensitive. BLCA, bladder urothelial carcinoma; BRCA, breast invasive carcinoma; COAD, colon adenocarcinoma; GBM, glioblastoma multiforme; HNSC, head and neck squamous cell carcinoma; KIRC, kidney renal clear cell carcinoma; LUAD, lung adenocarcinoma; LUSC, lung squamous cell carcinoma; OV, ovarian serous cystadenocarcinoma; READ, rectum adenocarcinoma; UCEC, uterine corpus endometrial carcinoma.

Next, we identified classes of solid tumors that were most sensitive to TLT using an unbiased computational approach. The gene-expression profiles of cancer cells are a valuable tool in the comprehension of transcriptional changes indicative of treatment. These profiles enable the identification of drug sensitivity across various cancer types. This approach has been used multiple times to show a pathway or drug sensitivity [34,35,36]. Accordingly, we generated a TLT sensitivity signature that reflected the genomic changes in eight TLT-sensitive BL-CL cell lines (Figure 5A). We treated the cell lines with either TLT or DMSO control and used RNA-sequencing to profile the samples. We identified 131 genes that were significantly upregulated or downregulated and incorporated the expression values for these genes into a predictive signature (Figure 5A). Then, we used this signature to predict drug sensitivity for the tumors from the PANCAN12 gene-expression dataset, which contains expression profiles for 12 cancer types [37,38]. The outcome of this process is a probability for each tumor sample, indicating how likely each tumor responds to TLT treatment. Breast cancer (mean = 0.71) and bladder cancer (mean = 0.69) were predicted to be most sensitive to TLT (1.00 is highest possible sensitivity, and 0.00 is lowest), while glioblastoma was predicted to be least sensitive (mean = 0.16, Figure 5B). These results are in line with the in vitro observations of TLT effectiveness against breast cancer (BL-CL breast cancers in particular) as well as bladder cancer. This is in agreement with recent data suggesting that some bladder cancers show marked similarities to CL breast cancer [39,40] and validates this prediction.

## 3. Discussion

We have identified and described a previously uncharacterized trisulfated sterol that we have named topsentinol L trisulfate (TLT) that exhibited increased tumoricidal activity against BL-CL breast cancers. Interestingly, halistanol sulfate, another trisulfated sterol isolated from the same marine organism as TLT, did not exhibit similar selective activity against BL-CL breast cancers but was demonstrated to be more potent (Figure S5). This could potentially be attributed to the differences in the side chains of these two compounds, which are otherwise structurally identical (Figure 2). TLT is not a good candidate for drug development due to its low potency and efficacy; however, it revealed novel targets to consider, in the future, for BL-CL breast cancers.

Furthermore, we describe the treatment effect of TLT on BL-CL breast cancers, highlighting the particular changes in the phosphorylation of AMPK, CHK1, and p38. AMPK is a heterotrimeric serine/threonine kinase complex that is regulated by adenylate levels in the cell and functions as part of an evolutionarily conserved energy-sensing pathway [41,42]. The effective result of AMPK activation is the avoidance of bioenergetic catastrophe and cell death through the conservation of cellular energy [42]. Interestingly, the role of AMPK in cancer is complex, as AMPK can exert pro- or antitumor effects based on cell context. AMPK is central to a tumor suppressor network, the LKB1-AMPK-TSC-mTOR signaling cascade, known to regulate cell growth and proliferation in response to stress [43]. Conversely, retaining continuous activation of AMPK leading to an enhanced ability to adapt to metabolic stress may function to promote tumor survival and growth. For example, the activation of AMPK in response to stresses such as hypoxia and nutrient deprivation provides cancer cells with the metabolic flexibility needed for survival [42]. These duelling roles of AMPK highlight the complexity and dichotomy of the kinase’s role in cancer cells. AMPK agonists acting as anticancer agents have been suggested through the use of the therapeutic biguanides, metformin, and phenformin. Metformin is currently used to treat type II diabetes and has been associated with a significantly lower cancer incidence in patients relative to those using other medications to manage their diabetes [32,44]. However, recent work has indicated that the anti-tumorigenic effects of metformin and another known AMPK agonist, AICAR, are due to AMPK-independent effects [31]. Interestingly, other studies have implicated AMPK as a mediator of cellular proliferation and survival, showing the promising effect of AMPK inhibition as a cancer therapy [45,46]. We observe similar effects against breast cancer, and particularly the BL-CL subtypes that exhibit higher sensitivity against dorsomorphin than luminal/HER2+ breast cancer (Figure 4A). This observation is in line with the inhibitory effects on AMPK in BL-CL breast cancers induced by TLT treatment (Figure 3A,B).

Another aspect of the inhibitory effects promoted by TLT treatment was the decreased phosphorylation of CHK1 leading to its inhibition. Upon cellular exposure to various genotoxic stresses, CHK1 is activated by ATR-mediated phosphorylation following DNA-damage leading to the phosphorylation of cdc25. CHK1 assumes the role of the major cell-cycle checkpoint kinase mediating S- and G2-arrest [28]. In BL-CL breast cancers, the rationale of CHK1 targeted therapy is supported by the documented evidence of alterations in the DNA damage repair machinery through either the high rate of BRCA or p53 mutations [7,8,11]. Therefore, another loss of a DNA damage repair component may lead to the cell’s inability to properly fix chromosomal damage and enter apoptosis. Indeed, Albiges et al. showed that CHK1 was a potential therapeutic target in TNBC, with CHK1 inhibition observed to induce mitotic cell death in TNBC cell lines [28]. Our observation of increased sensitivity of BL-CL cells to CHK1 inhibition is concordant with the TNBC subtype (Figure 4B).

Similarly, we have shown that treating BL-CL breast cancers with TLT leads to the significant activation of p38 (Figure 3A and Table S4); p38 plays the role of a signal transduction mediator and is linked to inflammation, cell cycle, cell death, cell differentiation, senescence, and tumorigenesis [37]. While this study does not demonstrate that p38 is involved in apoptosis in these experiments, other studies have shown that the activation of p38 leads to apoptosis in various cells [47,48].

The inhibitory effects of TLT on AMPK and CHK1 shed light on the potential therapeutic benefit of AMPK or CHK1 inhibition on BL-CL activity. However, additional work is required to validate these findings. Although dorsomorphin is a potent AMPK inhibitor, studies have shown this compound to exhibit high affinity towards other proteins such as BMP and inhibit several other kinases at a Km lower than AMPK [49], prompting us to validate the role of TLT-mediated inhibition of AMPK via genetic means (Supplemental Figure S6). Furthermore, TLT also strongly inhibited AKT, MAPK, RAF, FAK, STAT3, p70S6K, FASN, and PDK1 (Table S4), of which all of these are likely to have strong inhibitory effects on cell viability, and therefore cannot be discounted to participate in the killing mechanism alongside AMPK and CHK1 inhibition. Our work highlights the effects of TLT on BL-CL activity and implicates several proteins involved in its downstream repercussions but does not address the specific mechanism of action leading to these effects.

## 4. Materials and Methods 

### 4.1. Cell Lines and Viability Measurement

Cell lines were obtained from ATCC and plated at 1500–2000 cells/well in 384-well plates in 5% FBS (Gibco/Life Technologies, Carlsbad, CA, USA) growth media and 1× antibiotic-antimycotic (Anti-Anti) (Gibco/Life Technologies, Carlsbad, CA, USA). Cancer cell lines were cultured and maintained in a humidified environment at 37 °C and 5% CO_2_ in their respective media. The detailed description of the cell lines used for each screen is available in Tables S1 and S2. Cells were treated for 72 h, after which cell viability and growth were measured using CellTiter-Glo (Promega, Madison, WI, USA). Cell viability scores were calculated by dividing the viability scores of the treatment by the control DMSO values.

### 4.2. Marine Invertebrate Compound Library (MICL) Screens 

For Screen 1, 2778 HP20 fractions of marine organisms from MICL were screened at a single dose (~1.5 µg/mL) against a panel of 16 (9 lung and 7 breast) cancer cell lines to determine their antitumor properties. We selected 107 fractions for further evaluation based on one or more of the following criteria: (1) all fractions with a standard deviation in viability of greater than 0.325, (2) lung selective fractions (25% or less viable cells in 3 or more lung cancer cell lines and 2 or fewer breast cancer cell lines), (3) breast selective fractions (25% or less viable cells after treatment in 3 or more breast cancer cell lines and 2 or fewer lung cancer cell lines), (4) generally active non-universally toxic fractions (25% or less viability in a minimum of 5 and maximum of 13 cell lines), (5) relatively less active fractions (40% or less viability in a minimum of 9 and maximum of 13 cell lines). For our second screen, we added an additional 85 HP20 fractions from MICL that were not included in the first screen but based on the chemo-taxonomic judgment of potential chemical similarity to the 107 fractions. Then, these 192 HP20 fractions were assayed at a single dose (~1.5 µg/mL) against a panel of 35 breast cancer and 37 lung cancer cell lines. This identified breast-selective fractions (fractions resulting in 25% or less cellular viability after treatment in 13 cell lines or more out of 35 breast cancer cell lines, and 12 cell lines or fewer lung cancer cell lines) and fractions effective against BL-CL breast cancers (BL-CL breast cancers vs. luminal/HER2+ breast cancer unpaired two-sample equal variance *t*-test < 0.05 with a positive average difference). Cell lines described as basal-like or claudin-low but being HER2+ were considered to be part of the luminal/HER2+ group. Liquid chromatography mass-spectrum fractionation of the anti-BL-CL fractions, following the MICL protocol [13,14], resulted in 20 subfractions each that were assayed for effectiveness against BL-CL cell lines in a panel of 33 breast cancer cell lines. Once a candidate subfraction was determined based on the results of all three screens, large scale isolation and the purification of the active compound of that fraction were pursued (Supplementary Materials). We estimated the purity of TLT to be approximately 99% pure based on the lack of unassigned peaks in the NMR and MS spectra.

### 4.3. Dose-Response Assays

Cell lines were plated as described above. TLT and halistanol sulfate were serially diluted 1:2 starting from 114.13 µM to the lowest dose of 3.57 µM and screened against a panel of 30 breast cancer cell lines. Dorsomorphin and Ly2603618 were serially diluted 1:3 starting from 90 µM to the lowest dose of 41.15 nM in RPMI media containing 5% FBS and 1x Anti-Anti and screened against a panel of 20 breast cancer cell lines, along with the combination treatment of dorsomorphin and Ly2603618. For the combination treatment, an equal molar concentration of each compound was used. Cell viability was measured, as described before. Doses were repeated in quadruplicates and averaged for a single value. The 50% effective concentration (EC50) values were calculated from dose-response curve data by plotting on GraphPad Prism 6.01 and using the equation, $\;Y=1/\left(1+10^{\left(\left(logEC50-X\right)*HillSlope\right)}\right)$ with a variable slope (Ymin = 0 and Ymax = 1). Plots were forced to start from the x-axis by plotting for an x-intercept point.

### 4.4. Reverse Phase Protein Array

Eight TLT-sensitive BL-CL cell lines (MDA-MB-157, MDA-MB-436, MDA-MB-468, MDA-MB-231, HCC38, HCC70, HCC1395, and HCC1143) were treated separately with TLT or DMSO at a concentration of 105 µM (approximate average EC75 across all 8 cell lines) for 6 h, after which they were lysed, according to the method detailed in supplementary methods. This dose was used to elicit a pronounced TLT response prior to observable cytotoxicity. Reverse-phase protein array was performed at the University of Texas MD Anderson Cancer Center by the functional proteomics RPPA core facility according to their described methods and protocol [24,50]. Two hundred and seventeen different antibodies of phosphorylated and non-phosphorylated proteins were stained and quantified (Table S4).

### 4.5. RNA Sequencing Data Acquisition 

The same eight TLT-sensitive BL-CL cell lines that were used in the reverse-phase protein array assay were treated separately with TLT or DMSO for 6 h, after which total RNA was extracted using the RNeasy Mini Kit (Qiagen, Venlo, Netherlands) with on-column digestion of the genomic DNA, as described in the manufacturer’s protocol. RNA sequencing was performed at the Huntsman Cancer Institute High Throughput Genomics Core Facility using 50-cycle, single-read sequencing (version 3) on an Illumina HiSeq instrument. To construct mRNA focused libraries from total RNA, the Illumina TruSeq RNA Sample Prep Kit (version 2) with oligo(dT) selection was used.

### 4.6. TLT Sensitivity Signature Generation and Analysis

To process the mRNA sequencing data, we used the TCGA mRNA-seq Pipeline [38]. RNA sequencing reads for the treated and control samples were aligned using MapSplice v12_07 [47], quantified using RSEM [50], and gene counts were normalized using upper quantile normalization. The raw and processed data are available in the Gene Expression Omnibus under the series accession number GSE142833. This was the same methodology used to normalize the PANCAN12 TCGA dataset, which we obtained from TCGA fully processed for use in this analysis [38]. To generate a TLT sensitivity signature, we used the DESeq2 package (version 1.4.5) in the Bioconductor framework (version 2.14.0, version 3.1.0 of R) to identify genes that were significantly deregulated (adjusted *p* < 0.05) between the treated and control samples [51,52]. One hundred and forty-six genes were found to be significantly deregulated, among which only 131 were found in the TCGA dataset. To use DEseq2, the reads had to be re-mapped using the Rsubread Bioconductor package. We used this package to map the reads to version hg19 of the human genome and to summarize the data to gene-level values [53]. We predicted TLT sensitivity for the PANCAN12 TCGA dataset [38] using the Bayesian binary regression algorithm version 2.0 (BinReg2.0) used as a MATLAB plug-in [54]. We used default parameters, except that our signature used 131 genes and one metagene. The probability output from the binary regression model was subtracted from one so that probabilities closer to one indicated a higher probability of sensitivity to the drug, as previously described [36]. Prior to making the predictions, the data were log2 transformed and DWD normalized [55] to reduce biases that could result from differences in batch processing and platforms.

### 4.7. Immunostaining

Four BL-CL cell lines sensitive to topsentinol L trisulfate (HCC70, HCC1143, MDA-MB-468, and MDA-MB-436) were treated with 105 µM of TLT in 5% FBS RPMI media and 1x Anti-Anti for 6 h. Proteins were extracted and Western blots were run with the following primary antibodies: β-actin (#3700, RRID:AB_2242334); β-tubulin (#2146, RRID:AB_2210545); pRaptor-S792 (#2083); pCdc25c-S216 (#9528, RRID:AB_2075150); pMAPKAPK-2-Thr334 (#3007, RRID:AB_490936); p38-T180/Y182 (#4511S, RRID:AB_2139682); pChk1-S317 (#12302, RRID:AB_2783865); pAMPKα-T172 (#2535, RRID:AB_331250). All antibodies were obtained from Cell Signaling Technology (Beverly, MA, USA).

### 4.8. Statistical Analysis

To identify the candidate fractions that were significantly more effective against BL-CL breast cancers than luminal/HER2+ breast cancer, a preliminary statistical analysis was performed using the unpaired two-sample equal variance *t*-test built into the Microsoft Excel program. Final statistical assessment was performed for the fractions from the sponge PNG07-3-073 by re-analyzing the statistical significance test based on the normality of the data. Gaussian distribution of the data was checked by using the following three tests built into the GraphPad Prism 6 software: the D’Agostino–Pearson omnibus test, the Shapiro–Wilk test, and the Kolmogorov–Smirnov test (with the Dallal–Wilkinson–Lilliefor corrected P value). Then, dot plots were created using GraphPad Prism 6.01 and a standard two-tailed Mann–Whitney U test was used, with the exception of the dot plot diagrams for halistanol sulfate, dorsomorphin, Ly2603618, and dorsomorphin + Ly2603618, where an unpaired *t*-test was used due to the normality of the data. To compare the difference of protein expression between treated cell lines and their DMSO controls, we used a two-tailed paired *t*-test.

## 5. Conclusions

Our work describes the projected efficacy of TLT against a variety of human tumors, highlighting the optimal effect of the compound against breast and bladder cancer. In a TCGA study characterizing the molecular landscape of urothelial bladder carcinoma, a p53 mutation rate of 49% was recorded in the samples tested [56], drawing a similarity to the common observation of p53 mutation in BL-CL cells. Remarkably, among the key pathway nodes deregulated in bladder cancer, the LKB1/STK11-TSC-mTOR node was among the most commonly deregulated. LKB1, the activator of AMPK, was recorded to contain copy number alternations (CNAs) in 11% of all cases. TSC1 and TSC2 recorded CNAs in 16% and 9% of all cases, as well as inactivating mutations in 8% and 2% respectively [56]. Thus, one hypothesis is that the inhibitory effect of TLT on CHK1 and AMPK (and subsequently the LKB1-AMPK-TSC-mTOR node) could lead to an equally effective response against bladder cancer. Further work is needed to elucidate the exact mechanism of action of TLT and its projected effectiveness against bladder cancer.

In this study, we discovered and identified a previously unreported sulfated sterol, TLT, and described its signaling effects on BL-CL activity. We described two potential therapeutic targets of BL-CL cell lines that can be exploited for the benefit of treatment efficacy. This work lays the groundwork necessary for the exploration of AMPK and CHK1 as potential targets of BL-CL treatment, with a need to further characterize and delineate the role of TLT as an investigational anti-BL-CL compound.

## Figures and Tables

**Table 1:** Evaluation of TLT cytotoxic response.

Cell Line	Gene Expression Subtype	Topsentinol L Trisulfate EC50 (µM)
BT20	Basal-like	>100
HCC1143	Basal-like	74
HCC1395	Basal-like	77
HCC1806	Basal-like	>100
HCC1937	Basal-like	>100
HCC70	basal-like	23
MD468	Basal-like	69
HCC38	Claudin-low	>100
Hs578T	Claudin-low	99
MD157	Claudin-low	50
MD231	Claudin-low	34
MD436	Claudin-low	87
HCC1569	Basal/HER2 positive	>100
HCC1954	Basal/HER2 positive	63
JMT-1	Basal/HER2 positive	41
MD453	Luminal	>100
HCC2218	Luminal	>100
Cama-1	Luminal	>100
MD134VI	Luminal	>100
MD175VII	Luminal	>100
MD415	Luminal	>100
ZR75-1	Luminal	>100
MCF7	Luminal	>100
T47D	Luminal	26
BT483	Luminal	>100
AU565	Luminal/HER2 positive	>100
HCC1419	Luminal/HER2 positive	>100
SKBR3	Luminal/HER2 positive	>100
BT474	Luminal/HER2 positive	84
UACC812	Luminal/HER2 positive	>100

## Data Availability

The data presented in this study are openly available in raw and processed form in the Gene Expression Omnibus under the series accession number GSE142833.

## Supplementary Materials

The following are available online at https://www.mdpi.com/1660-3397/19/1/41/s1.
