# Supplementary material for: Topsentinol L Trisulfate, a Marine Natural Product That Targets Basal-like and Claudin-Low Breast Cancers
Source: Mar Drugs. 2021 Jan 18;19(1):41. doi: 10.3390/md19010041 (PMC7831112; doi:10.3390/md19010041)
Supplement: Supplementary file 1 [file marinedrugs-19-00041-s001.pdf]

Supplementary Material for

Topsentinol L Trisulfate, a new Marine Natural Product, that Targets Basal-like and Claudin-low Breast Cancers

Authors: Nader N. El-Chaar, Thomas E. Smith, Gajendra Shrestha, Stephen R. Piccolo, Mary Kay Harper, Ryan M. Van Wagoner, Zhenyu Lu, Ashlee R. Venancio, Chris M. Ireland, Andrea H. Bild, Philip J. Moos

1 Supplementary Methods

1.1 Small Molecules

Dorsomorphin and Ly2603618 were purchased from Selleckchem and dissolved in water and 100% DMSO respectively. Dorsomorphin was prepared as a stock of 100 mM, while Ly2603618 was prepared as a 28.65 mM stock.

1.2 Adenoviruses

CHK1 adenovirus was purchased from Vector Biolabs (Philadelphia, PA). GFP adenovirus was previously described (1). AMPK (constitutively-active) was a generous donation from Dr. Kenneth Walsh at Boston University.

1.3 Large-scale Isolation and Purification of Active Compound

Frozen PNG07-3-073 *Topsentia* sp. sponge (215 g) was extracted three times with methanol. One half of the pooled methanol extract was fractionated on Diaion HP20SS resin, eluting with water/isopropanol mixtures in 25% increments, followed by 100% methanol to yield five fractions, FW and F1-F4, where F2 was the only fraction that exhibited tumoricidal properties. F2 (50/50 IPA/H_2O, 98 mg) was subjected to C_{18} flash chromatography, eluting with 40-100% methanol in water in 10% increments, yielding 46 subfractions, of which only subfractions 22, 23, 24 and 25 showed tumoricidal activity. Bioactive fractions 22-25 were combined (65 mg) and further fractionated by HPLC using a Luna 5 µm phenylhexyl column (250 x 4.6 mm) and a gradient of acetonitrile (ACN) with 0.2 M NaCl (36% ACN increasing over 4 min to 43% ACN, held for 11 min, then decreased to 36% ACN over 2 min) to yield isolate 1 (sharp peak, RT=9.57 min, 13.4mg) and isolate 2 (broad peak, RT=10.2 min, 6.3mg). Salt was removed by loading samples onto pre-equilibrated Waters Sep-Pak C18 cartridges and flushing with three column volumes of 10% methanol, followed by compound elution with 100% methanol. The structures of the compounds were defined using MS and NMR with the primary changes being resonances for the side chain. In particular, the structure of isolate-1 was determined by NMR analysis to be identical to halistanol sulfate and was verified by HRESIMS (*m/z* = 607.2958 $[M\text{-}SO_3\text{-}H]^-$, calculated for $C_{29}H_{51}O_9S_2$, 607.2980, Δ = -3.9 ppm) (2). Analysis of spectral data for isolate 2 indicated that it had the same structure as topsentinol L (3) with sulfate groups located at positions C-2, C-3, and C-6. We have named this compound topsentinol L trisulfate (TLT): White solid, $[\alpha]^{20}_D$ +12.0 (c 0.212, MeOH); UV (MeOH) λmax (log e) 215 nm (2.98); 1H and 13C NMR (Supplemental Table 3); high-resolution electrospray ionization mass spectrometry (HRESIMS) m/z 619.2971 $[M–SO_3–H]^-$ (calculated for C30H51O9S2, 619.2980; Δ -1.5 ppm).

1.4 Cell Lysis and Western Blotting

Cell lines were washed once with ice-cold PBS (1x) and then lysed in a buffer containing 1% Triton X-100, 50mM HEPES, pH 7.4, 150mM NaCl, 1.5mM $MgCl_2$, 1mM EGTA, 100mM NaF, 10mM Na pyrophosphate, 1mM Na_3VO_4, 10% glycerol, and protease inhibitors (P8340) added fresh from Sigma-Aldrich (St Louis, Missouri). Lysates were then incubated on ice for 15 minutes with agitation every two minutes and then centrifuged at 14,000 RPMs in 4 °C. Protein yield was quantified using the Bradford reagent (Amresco, Solon, OH) and denatured by boiling and SDS treatment (with beta-mercaptoethanol). Equivalent amounts of protein (approximately 30 µg) were resolved on 12% precast SDS-PAGE gels (Biorad, Hercules, CA) and transferred to Immun-blot PVDF membrane (Biorad, Hercules, CA). IgG HRP-linked Secondary antibodies were used from GE Lifesciences (Pittsburgh, PA). Western blots were developed using SuperSignal West Dura Substrate (Thermoscientific, Rockford, IL).

1.5 Adenovirus-infected Dose Response Assays

Cell lines HCC1143 and MDA-MB-436 were infected with adenovirus expressing GFP, AMPK, CHK1, or with AMPK and CHK1 adenoviruses simultaneously with a multiplicity of infection (MOI) of 150 and left overnight. Cell lines were then plated 1000-2000 cells/well in 384-well plates in 5% FBS and treated with TLT for 72 hrs. TLT was dosed by serially diluting 1:2 starting from 104.4 µM to the lowest dose of 3.26 µM. Cell viability was measured as described in the “Cell lines and Viability Measurement” subsection. Doses were repeated in quadruplicates and averaged out for a single value. Dose-response curves were plotted and EC50 values calculated as described in the “Dose-Response Assays” subsection.

2 Supplementary Figures and Tables

2.1 Supplementary Figures

Supplementary Figure 1: Image of the marine invertebrate *Topsentia* sp. sponge labeled PNG07-3-073. The sponge specimen PNG07-3-073 was identified as *Topsentia* sp. (OTU UU1273), order Halichondrida, family Halichondriidae. The creamy white sponge is massive with thick fistular processes, a firm texture, and a slightly hispid surface. The ectosome is not detachable, consisting of a thin paratangential layer of spicules with protruding choanosomal spicules. The interior of the sponge is densely spiculous and completely disorganized with plumose bundles of spicules just below the surface, spongin not visibly present. The spicules are fusiform oxea in a range of sizes and widths, generally in categories: ~1125-1325 × 42-48µm, 715-1050 × 25µm, 525-825 × 15µm. *Topsentia* sp. (OTU UU1273) does not compare entirely to previously described species of *Topsentia* and likely represents an undescribed species. The sponge was collected by SCUBA November 21, 2007 from New Britain, Papua New Guinea (S 05° 19.680’, E 150° 17.674’) and immediately frozen. **The specimen has been described in** SpongeMaps, available at the following link: http://www.spongemaps.org/#!Topsentia-sp-OTU-UU1273-demosponge/zoom/c21kz/imagel3v

Supplementary Figure 2: 1D NMR spectra for halistanol sulfate and topsentinol L trisulfate in CD_3OD. 1D NMR spectra for halistanol sulfate in CD_3OD, **(A)** ^1H spectrum **(B)** ^{13}C spectrum, and topsentinol L trisulfate in CD_3OD, **(C)** ^1H spectrum **(D)** ^{13}C spectrum.

Supplementary Figure 3: Low-resolution mass spectra for halistanol sulfate and topsentinol L trisulfate. Low-resolution mass spectra for **(A)** halistanol sulfate and **(B)** topsentinol L trisulfate.

Supplementary Figure 4: 2D NMR spectra for topsentinol L trisulfate in CD_3OD. 2D NMR spectra for topsentinol L trisulfate in CD_3OD, **(A)** gCOSY spectrum, **(B)** zTOCSY spectrum, **(C)** ROESY spectrum, **(D)** gHSQC spectrum, **(E)** gHMBC spectrum.

Supplementary Figure 5: Halistanol sulfate is not selective against BL-CL in comparison to Luminal/HER2+ breast cancer. The cell lines of the investigated breast cancer panel were divided accordingly and scatter dot plot diagrams of halistanol sulfate EC50 values plotted. Every dot represents a cell line, with the y value representing the cell line's halistanol sulfate compound EC50 and the horizontal line indicating the mean value for every group. There is no significant difference between the response of BL-CL and Luminal/HER2+ cell lines to halistanol sulfate.

Supplementary Figure 6: Overexpression of AMPK and CHK1 individually or concurrently sensitizes BL-CL cell lines to TLT treatment. BL-CL cell line **(A)** HCC1143 and **(B)** MDA-MB-436 were infected with adenovirus vectors expressing GFP, CHK1, AMPK or AMPK+CHK1 with a multiplicity of infection (MOI) of 150 overnight. Afterward, a dose-response assay with TLT was performed and response curves plotted. The x-axis indicated the log10 dose of TLT. Y-axis indicates the percent response as compared to vehicle control (DMSO). The more the curves are skewed to the left, the more sensitive they are to TLT treatment. Green = GFP, Blue = CHK1, Black = AMPK+CHK1, Red = AMPK.

2.2 Supplementary Tables

Supplementary Table 1: Characteristics of the breast and lung cancer cell lines used in the study for all screens and drug response assays. The characteristics of all the cell lines used in this study are described in this table. For lung cancer cell lines, a description of EGFR, p53 and KRAS mutations exists. This information is portrayed by the specific amino acid mutations and frameshift deletions observed in their respective proteins where "*" denotes a stop codon.

Supplementary Table 2: List of cell lines used for each drug screen. Cell lines that were part of particular screens have an "x" in the corresponding screen column.

Supplementary Table 3: ^{13}C and ^{1}H chemical shifts of halistanol sulfate and topsentinol L trisulfate in CD_3OD. δ_H (500 MHz) and δ_C (125 MHz) NMR data for halistanol sulfate and topsentinol L trisulfate in CD_3OD**.**

Supplementary Table 4: RPPA results of the 8 BL-CL cell lines treated with DMSO or TLT. The RPPA normalized linear results of all the 217 proteins screened post-DMSO or TLT 6 hrs treatment. The scores represent normalized linear values. Pink indicates proteins significantly deregulated by TLT treatment (Paired t-test). Orange indicates proteins exhibiting a downregulation or upregulation of 15% or more. 21 proteins exhibited a 15% or more upregulation or downregulation in protein level (orange), with only eight of these proteins exhibiting statistically significant changes (orange and pink)**.**

Supplementary Table 5: TLT EC50 decreases with overexpression of CHK1 and/or AMPK. A table summarizing TLT EC50 (µM) is provided for each cell line following adenovirus-mediated overexpression of GFP, AMPK, CHK1 or AMPK+CHK1.

REFERENCES

1. Bild AH, Yao G, Chang JT, Wang Q, Potti A, Chasse D, et al. Oncogenic pathway signatures in human cancers as a guide to targeted therapies. Nature 2006;439(7074):353-7.

2. Fusetani N, Matsunaga S, Konosu S. Bioactive marine metabolites II. Halistanol sulfate, as antimicrobial novel steroid sulfate from the marine sponge Halichondria cf. moorei Bergquist. Tetrahedron Lett 1981;22(1985-1988).

3. Dai J, Sorribas A, Yoshida WY, Kelly M, Williams PG. Topsentinols, 24-isopropyl steroids from the marine sponge Topsentia sp. Journal of natural products 2010;73(9):1597-600.

Supplementary Figure 1

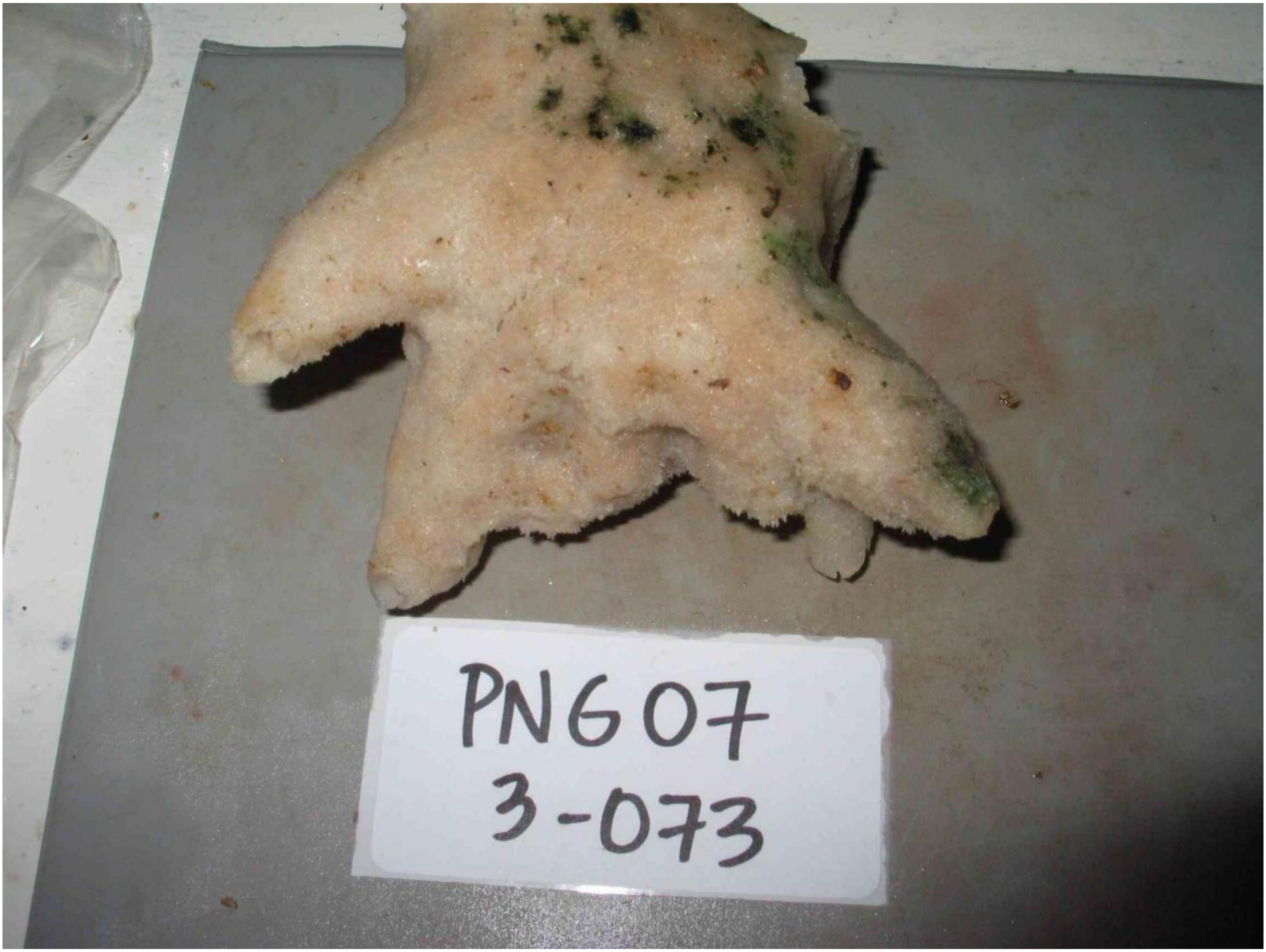

Supplementary Figure 2A

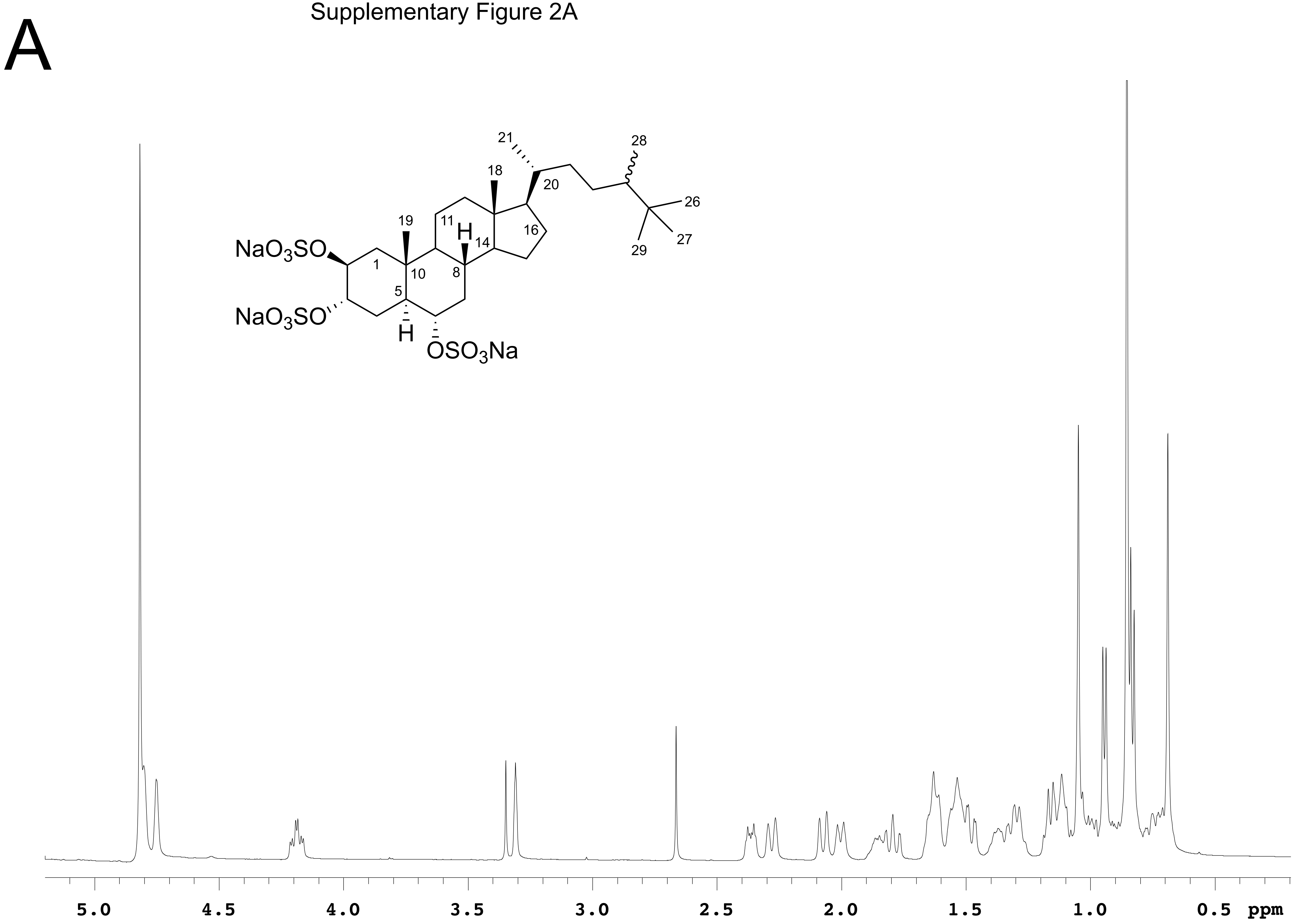

Supplementary Figure 2B

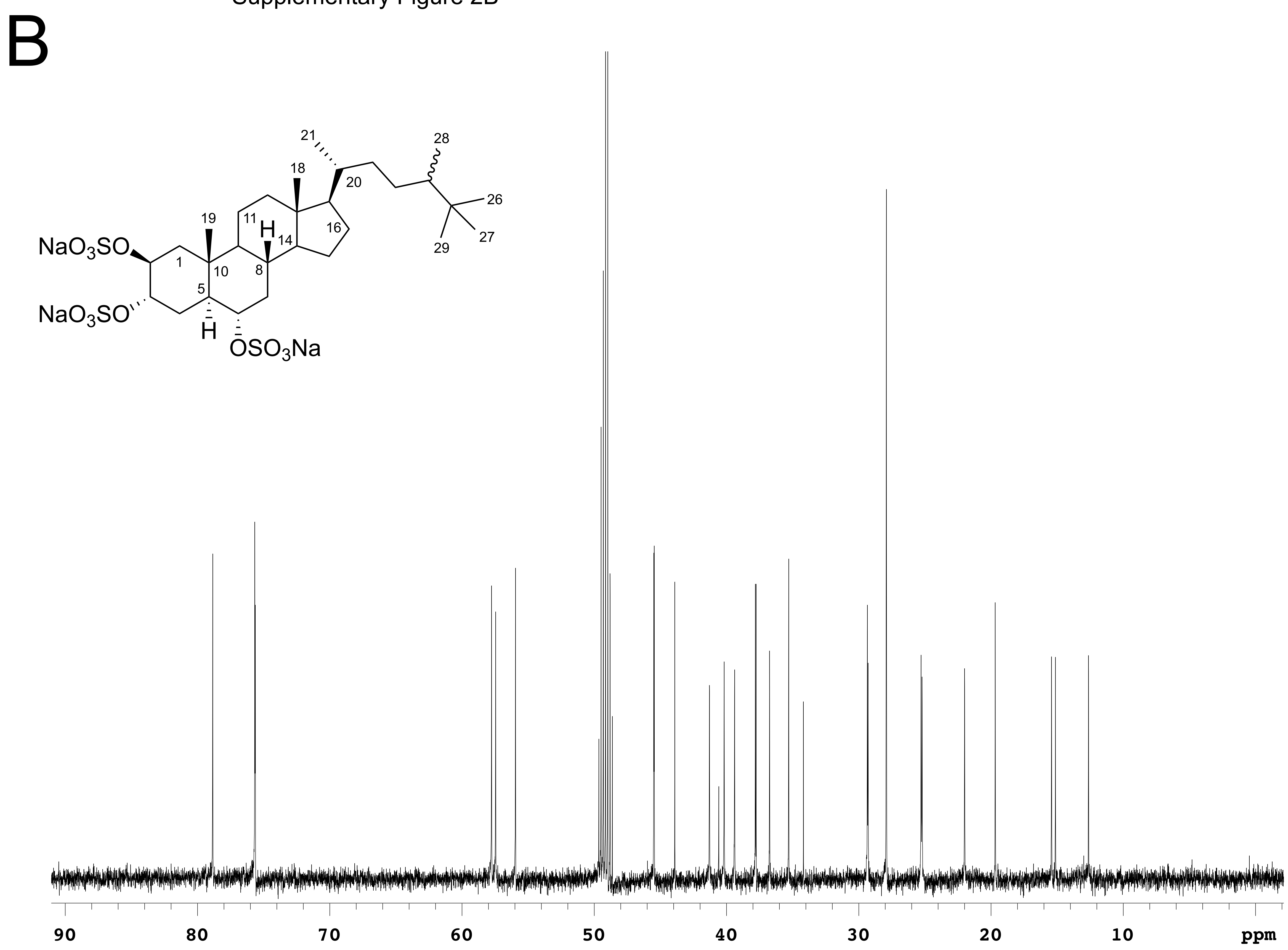

Supplementary Figure 2C

C

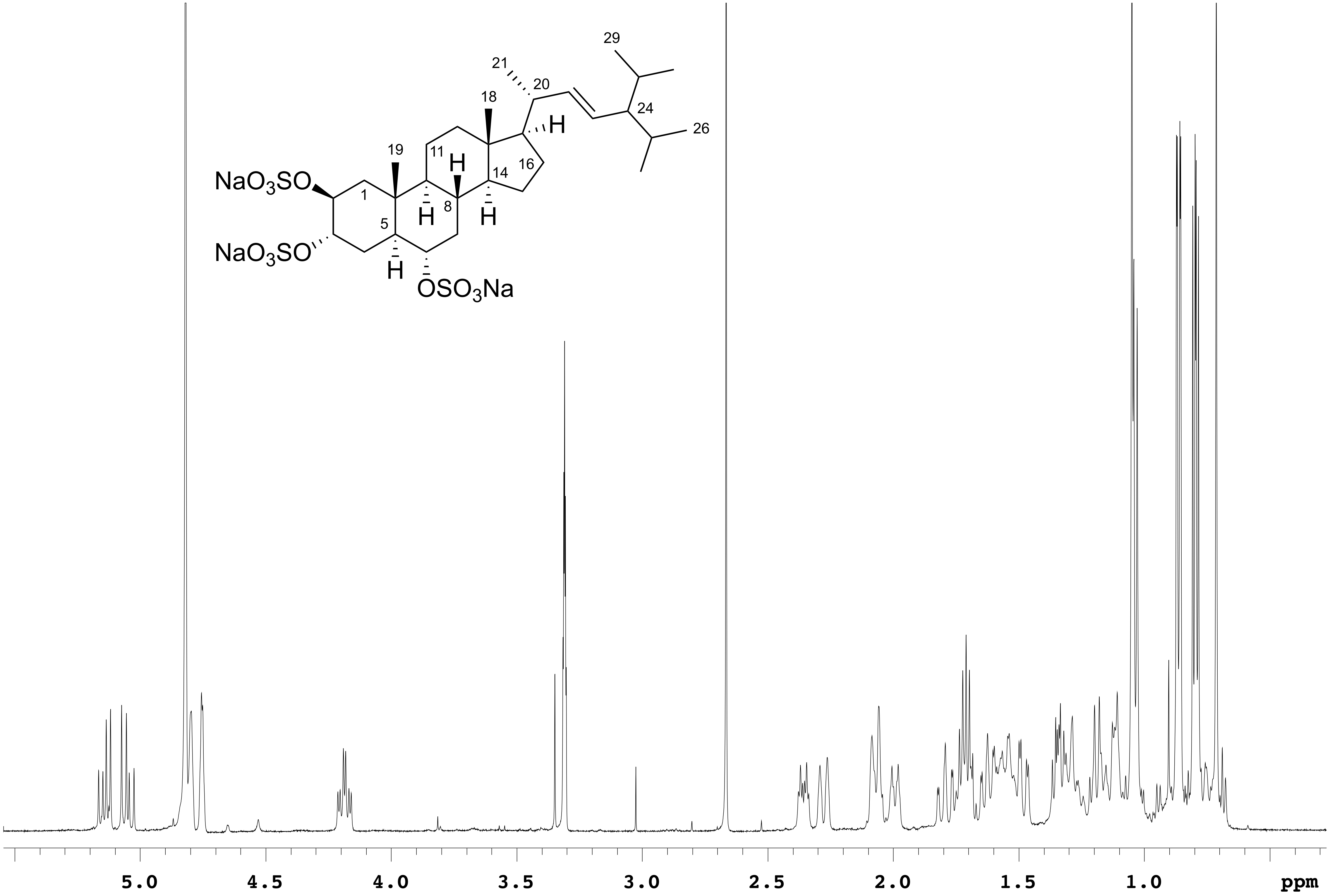

Supplementary Figure 2D

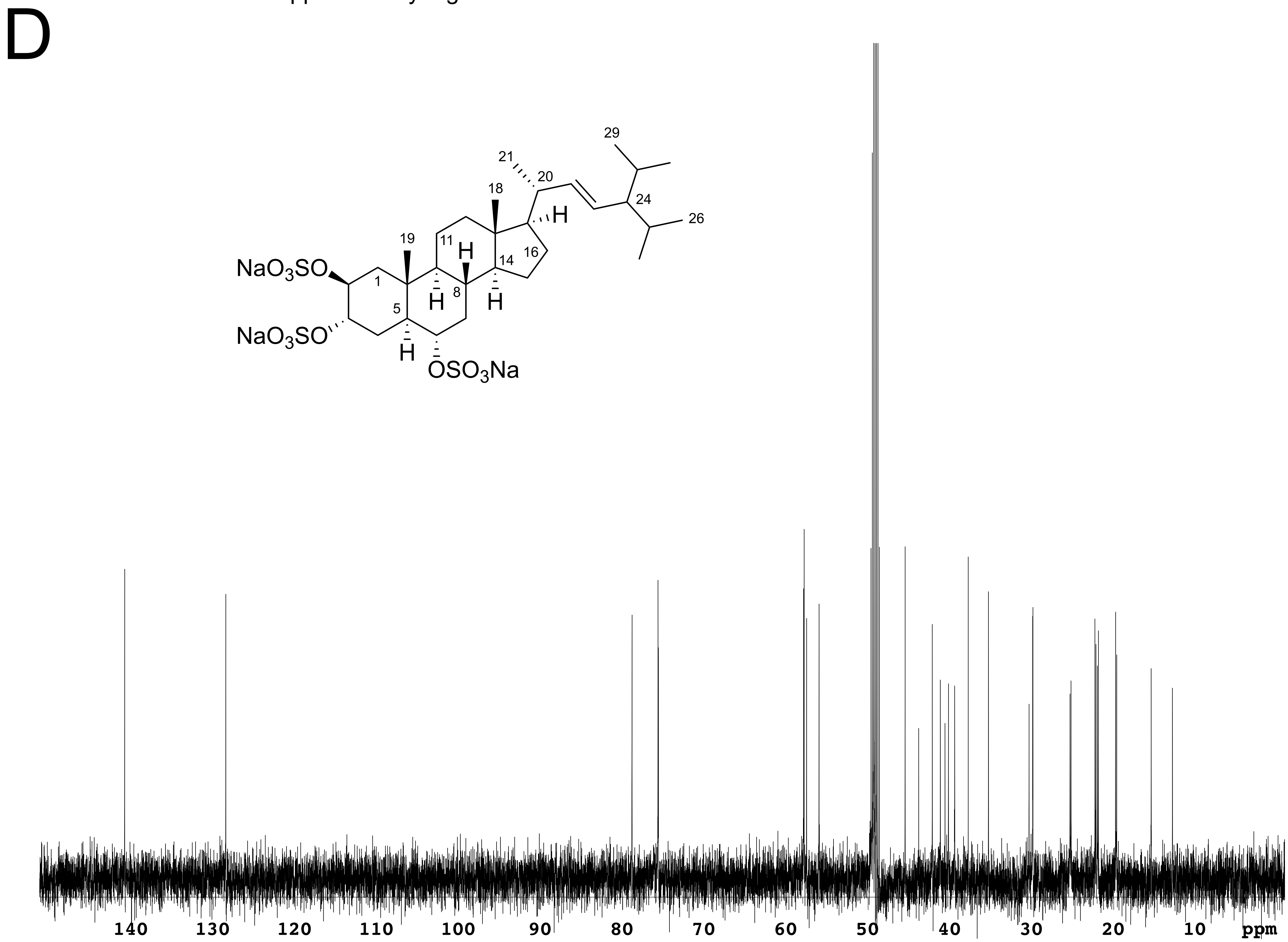

Supplementary Figure 3A

A

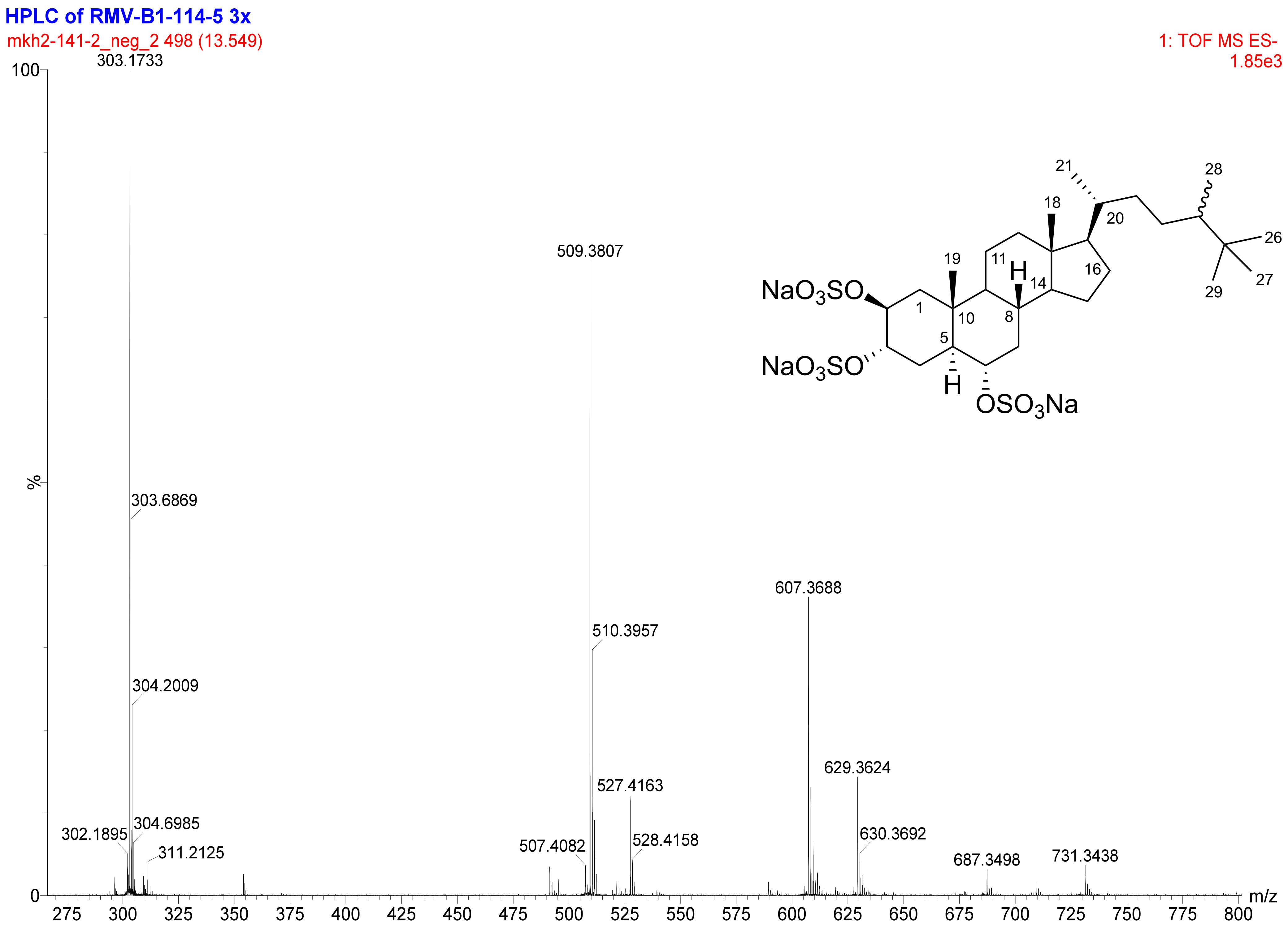

B

Supplementary Figure 3B

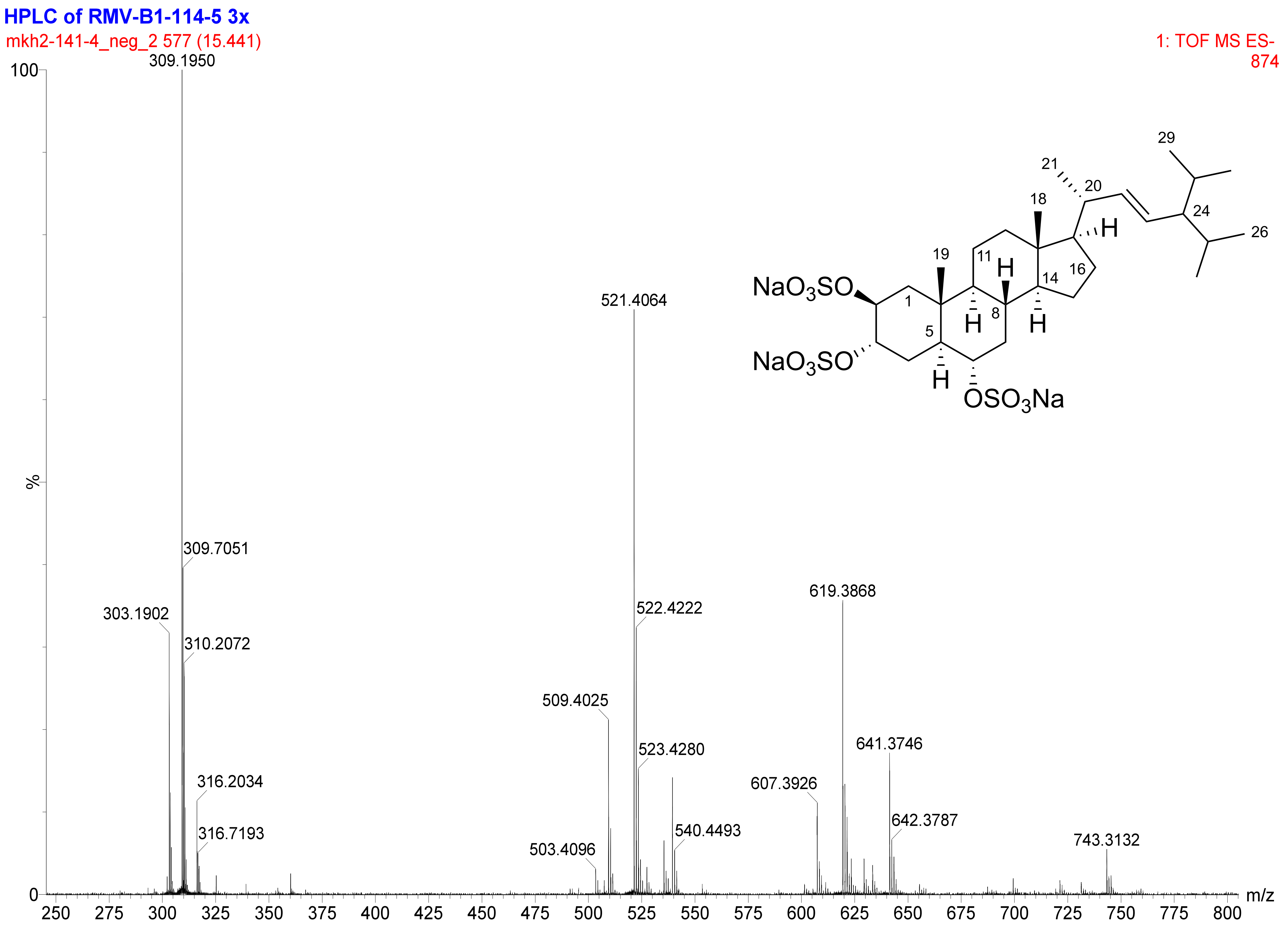

A

Supplementary Figure 4A

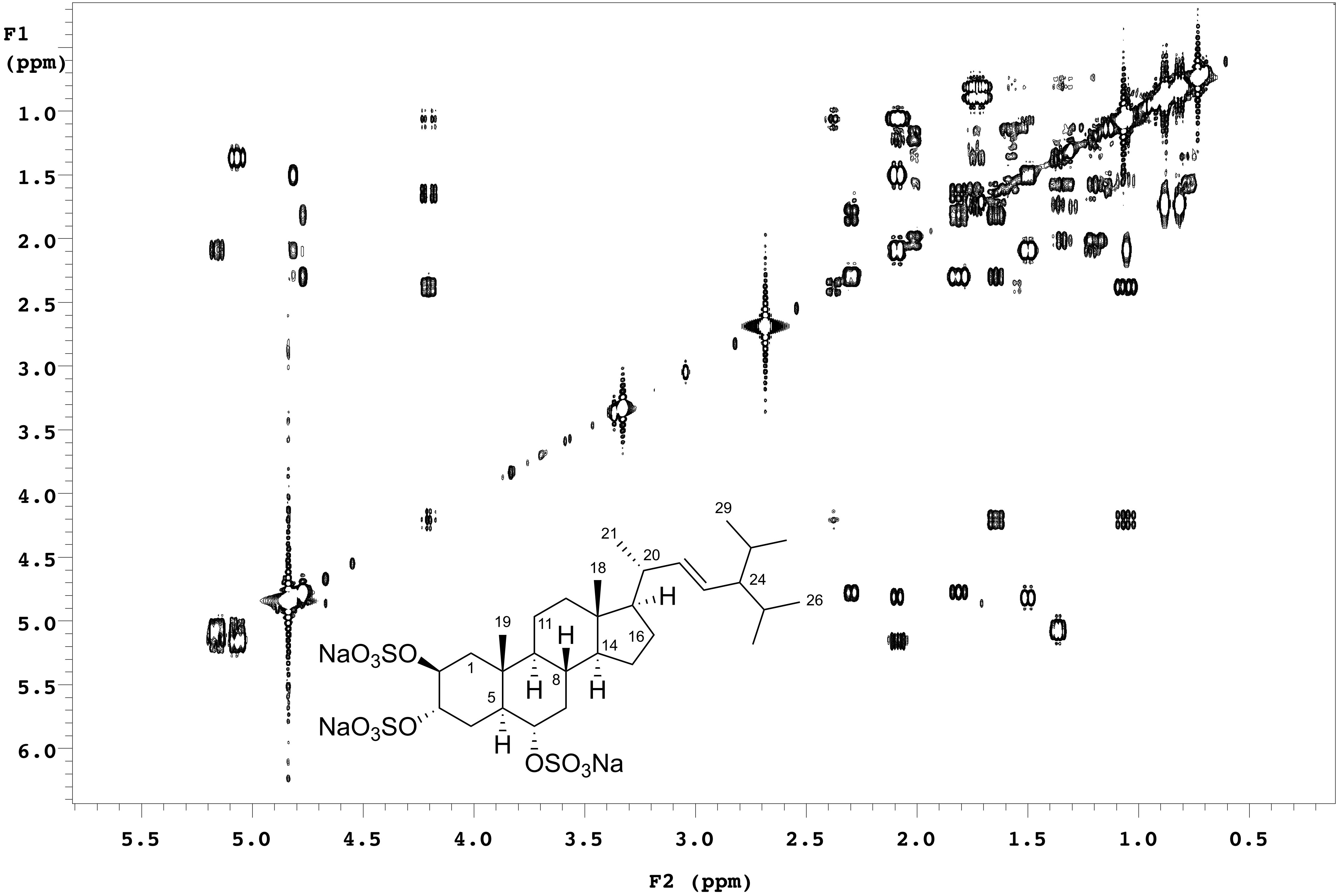

B

Supplementary Figure 4B

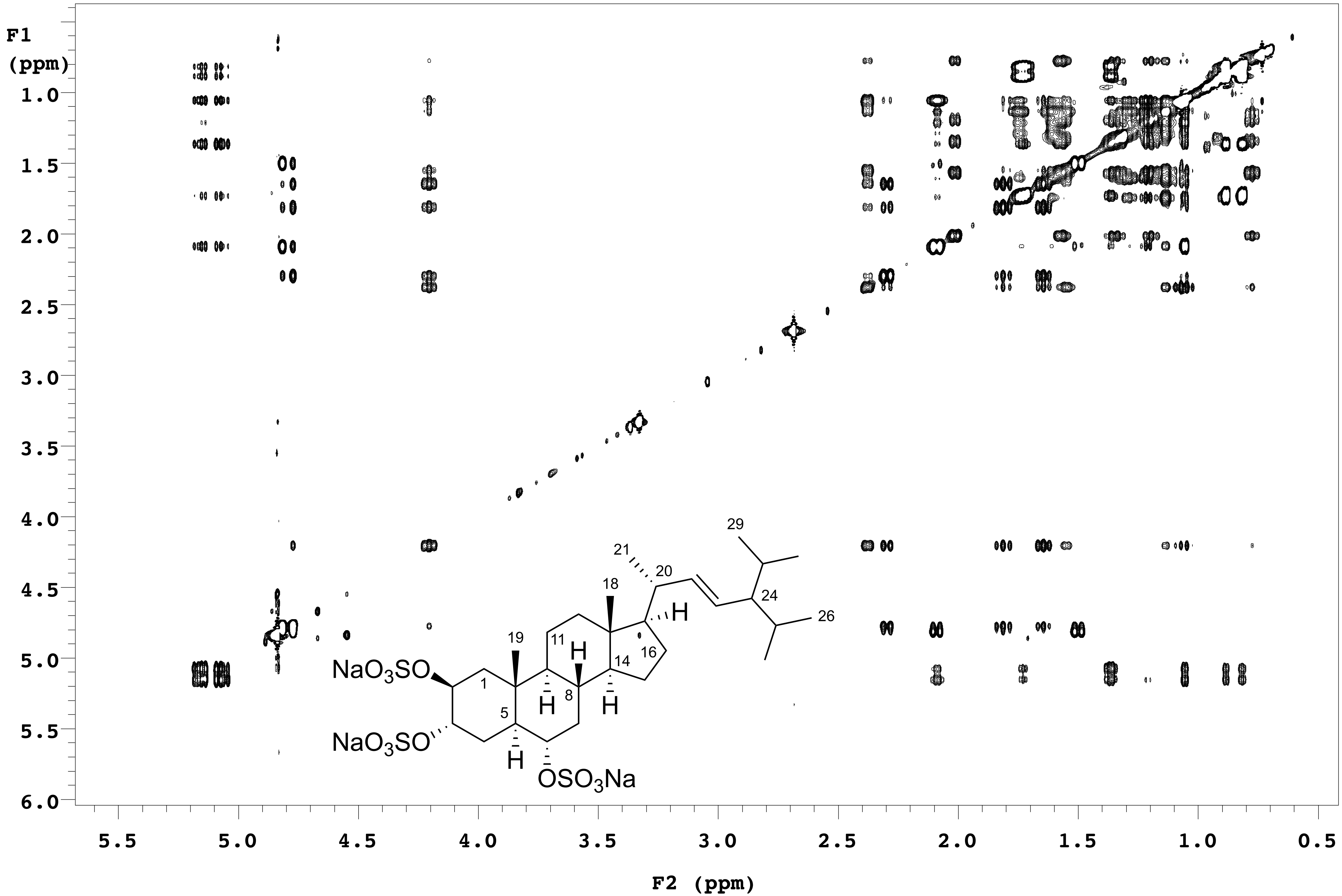

C

Supplementary Figure 4C

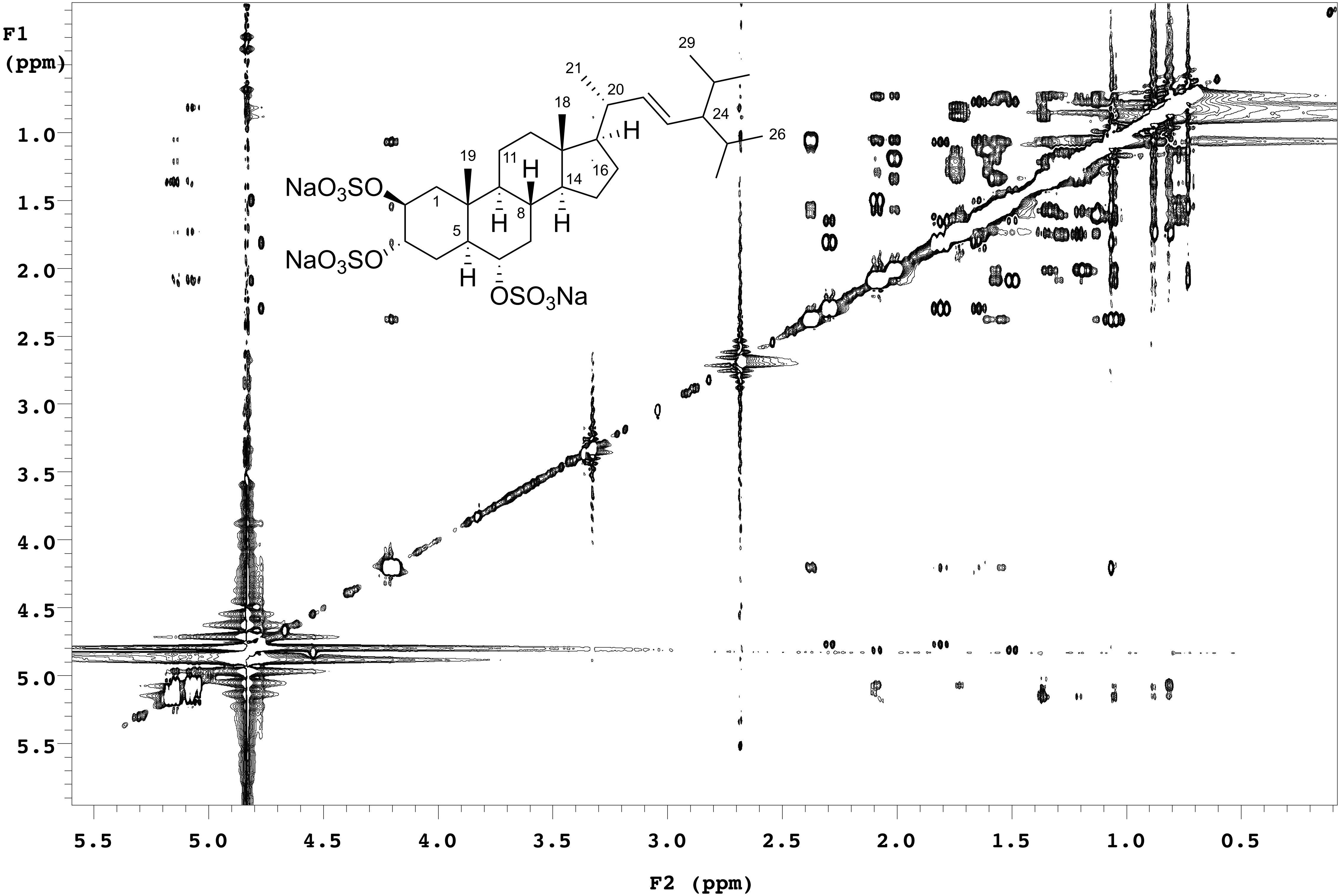

D

Supplementary Figure 4D

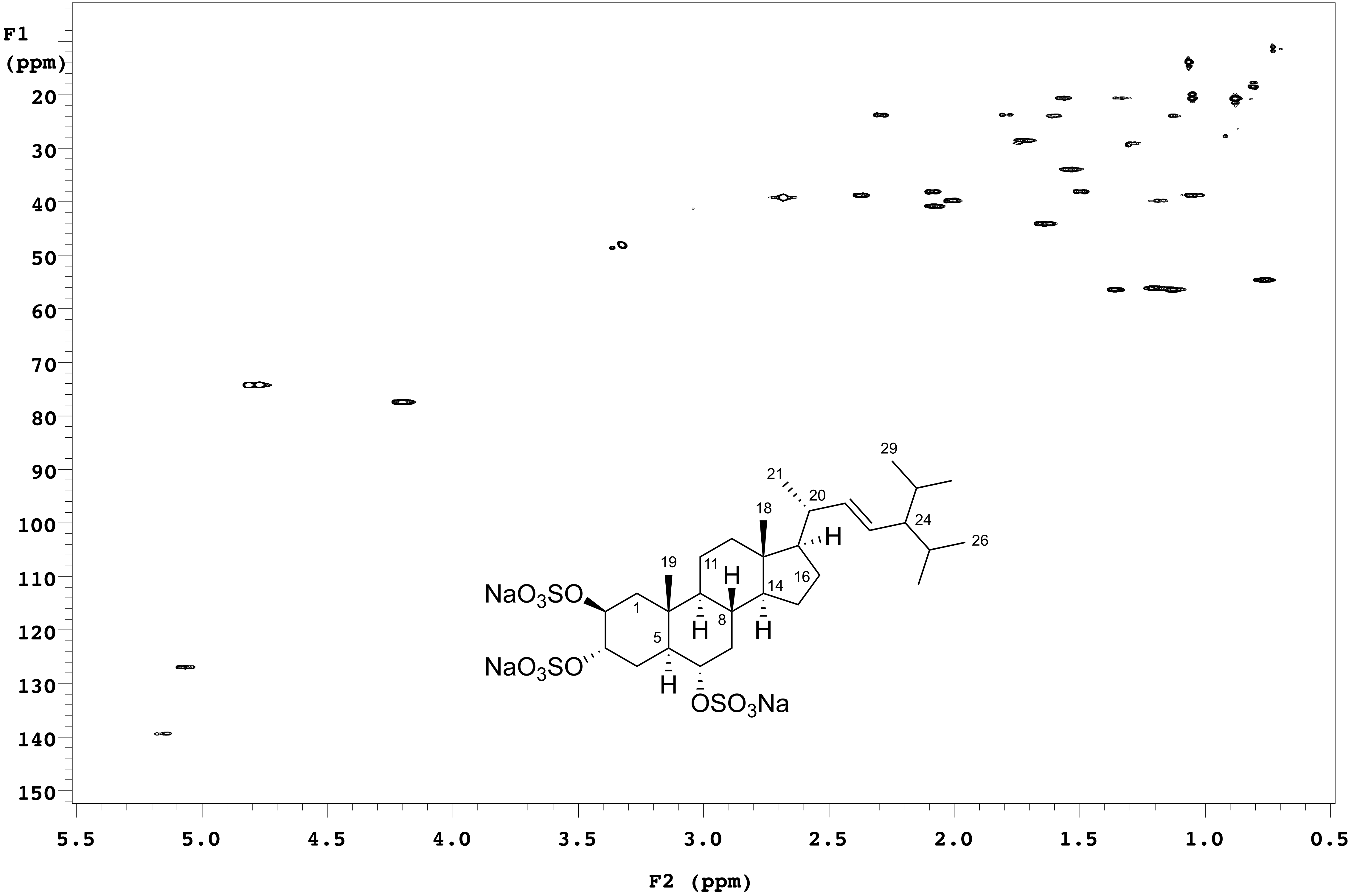

E

Supplementary Figure 4E

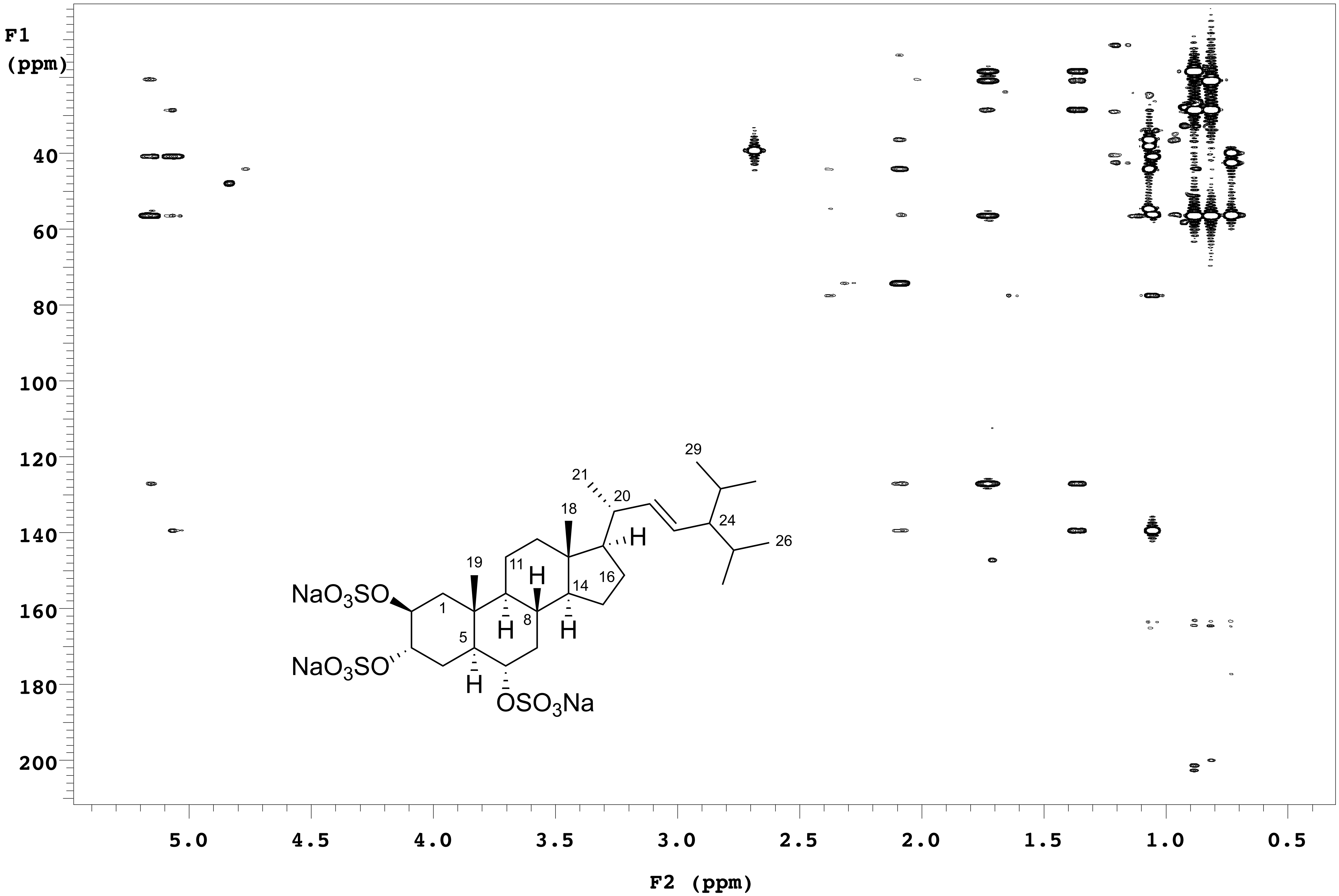

Supplementary Figure 5A

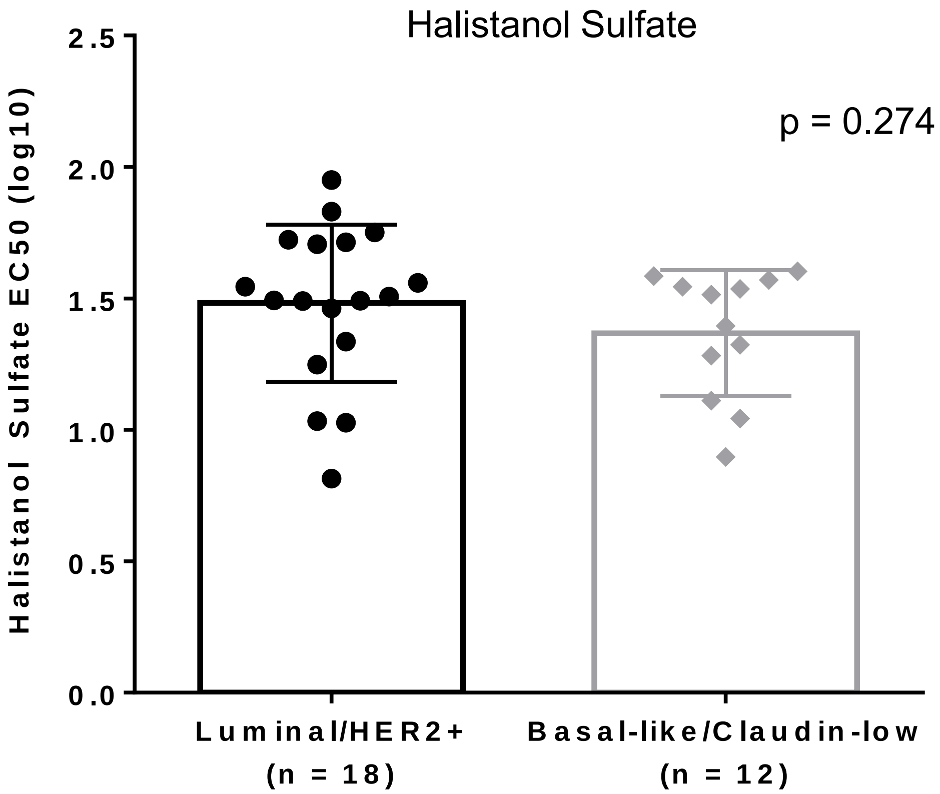

Supplementary Figure 5B

Topsentinol L Trisulfate

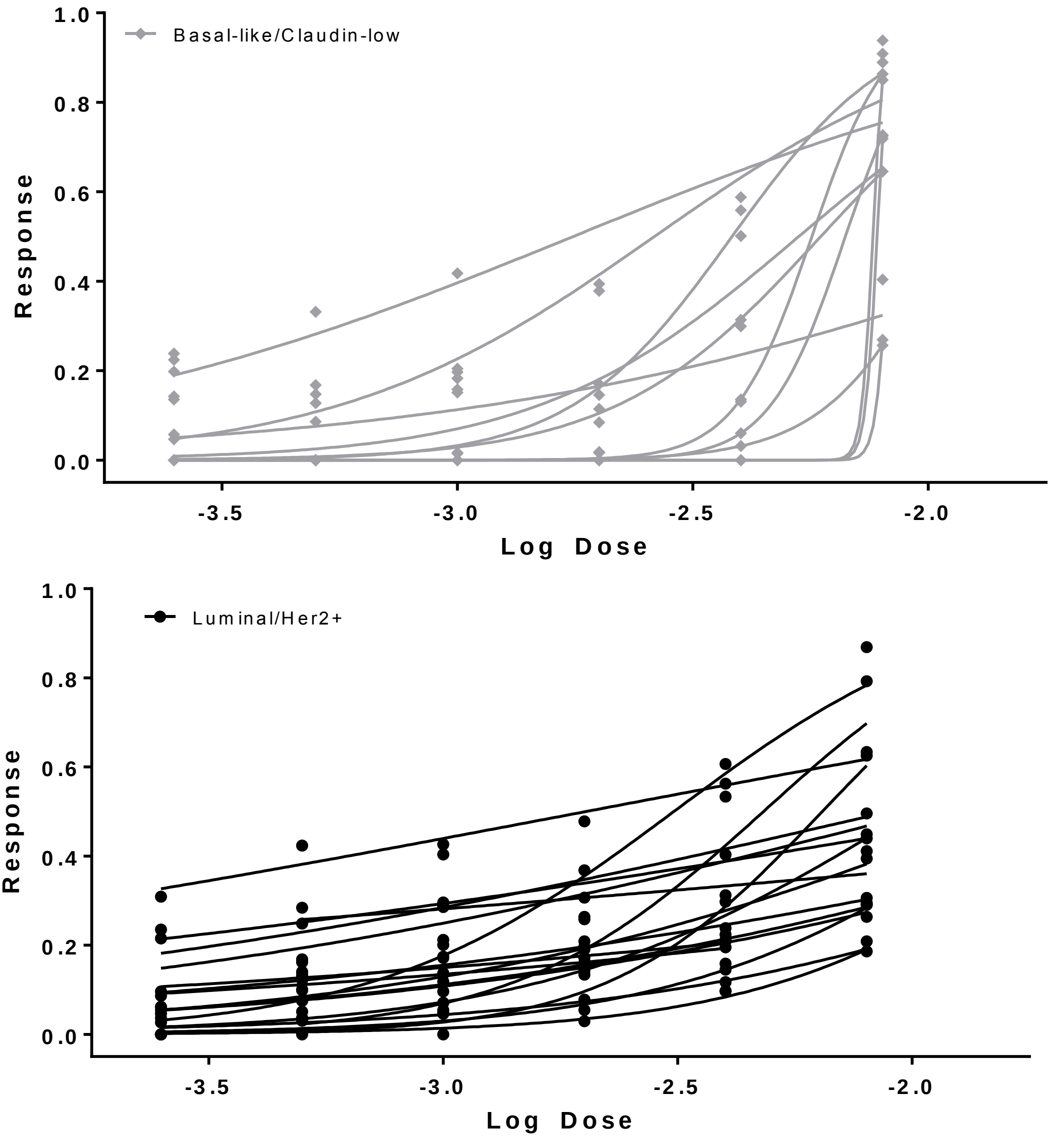

Supplementary Figure 6

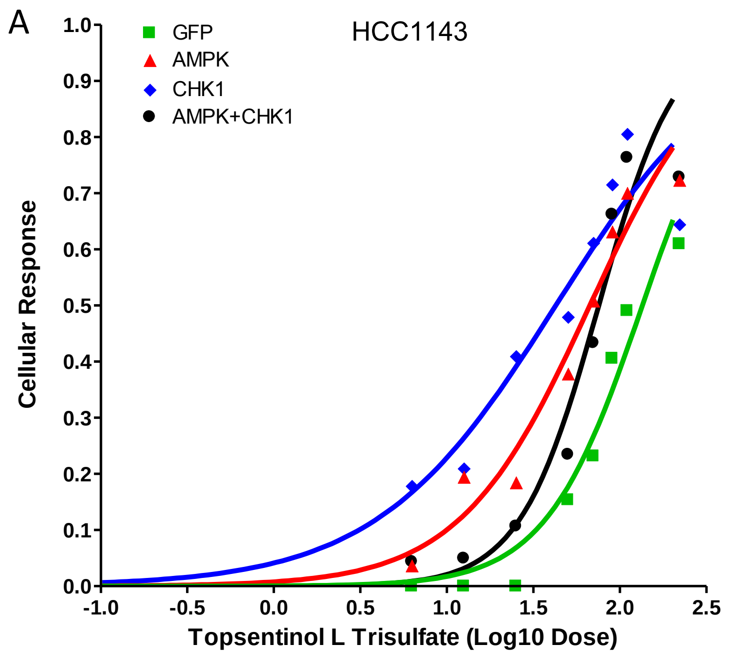

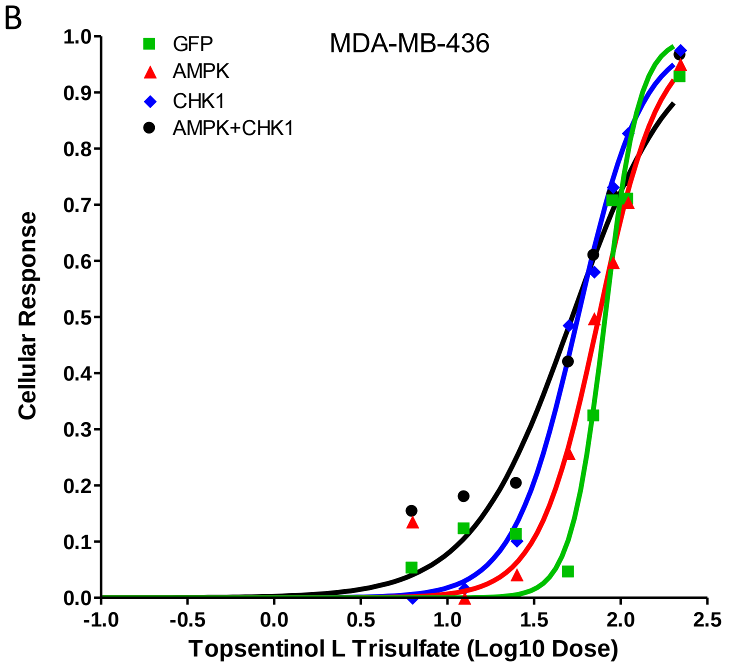

Supplementary Table 1

Breast Cancer Cell Line	ER	PR	HER2	Gene-expression Subtype	Media	ATCC Number
AU565	-	-	+	luminal/HER2 positive	RPMI	CRL-2351
BT-20	-	-	-	basal	DMEM	HTB-19
BT-474	+	+	+	luminal/HER2 positive	DMEM	HTB-20
BT-483	+	+	-	luminal	RPMI	HTB-121
BT-549	-	-	-	claudin low	RPMI	HTB-122
CAMA-1	+	-	-	luminal	DMEM	HTB-21
HCC1143	-	-	-	basal	RPMI	CRL-2321
HCC1395	-	-	-	basal	RPMI	CRL-2324
HCC1419	-	-	+	luminal/HER2 positive	RPMI	CRL-2326
HCC1500	+	+	-	basal	RPMI	CRL-2329
HCC1569	-	-	+	basal/HER2 positive	RPMI	CRL-2330
HCC1599	-	-	-	basal	RPMI	CRL-2331
HCC1806	-	-	-	basal	RPMI	CRL-2335
HCC1937	-	-	-	basal	RPMI	CRL-2336
HCC1954	-	-	+	basal/HER2 positive	RPMI	CRL-2338
HCC2218	-	-	+	luminal	RPMI	CRL-2343
HCC38	-	-	-	claudin low	RPMI	CRL-2314
HCC70	-	-	-	basal	RPMI	CRL-2315
Hs 578T	-	-	-	claudin low	DMEM	HTB-126
JIMT-1	-	-	+	basal/HER2 positive trastuzumab Res	DMEM	Unavailable
MCF7	+	+	-	luminal	DMEM	HTB-22
MDA-MB-134-VI	+	-	-	luminal	RPMI	HTB-23
MDA-MB-157	-	-	-	claudin low	DMEM	HTB-24
MDA-MB-175-VII	+	-	-	luminal	RPMI	HTB-25
MDA-MB-231	-	-	-	claudin low	RPMI	HTB-26
MDA-MB-415	+	-	-	luminal	RPMI	HTB-128
MDA-MB-436	-	-	-	claudin low	RPMI	HTB-130
MDA-MB-453	-	-	-	luminal	RPMI	HTB-131
MDA-MB-468	-	-	-	basal	RPMI	HTB-132
MDA-MB-361	+	-	+	luminal/HER2 positive	DMEM	HTB-27
SK-BR-3	-	-	+	luminal/HER2 positive	RPMI	HTB-30
T-47D	+	+	-	luminal	RPMI	HTB-133

Breast Cancer Cell Line	ER	PR	HER2	Gene-expression Subtype	Media	ATCC Number
UACC-812	-	-	+	luminal/HER2 positive	DMEM	CRL-1897
ZR-75-1	+	-	-	luminal	RPMI	CRL-1500
ZR-75-30	+	-	+	luminal/HER2 positive	RPMI	CRL-1504

Lung Cancer Cell Line	Subtype	K-Ras Mut	P53 Mut	EGFR Mut	Media	ATCC Number
A549	Adenocarcinoma	G12S	x	x	F12K	CCL-185
Calu-3	Adenocarcinoma	x	M237I	x	EMEM	HTB-55
NCI-H1155	Large cell	Q61H	R273H	x	ACL-4	CRL-5818
NCI-H1355	Adenocarcinoma	G13C	E285K	Q1159H	ACL-4	CRL-5865
NCI-H1373	Adenocarcinoma	G12C	E339*	Intron	RPMI	CRL-5866
NCI-H1395	Adenocarcinoma	x	x	x	RPMI	CRL-5868
NCI-H1437	Adenocarcinoma	x	R267P	x	RPMI	CRL-5872
NCI-H1563	Adenocarcinoma	x	x	x	RPMI	CRL-5875
NCI-H1581	Large cell	x	Q144*	x	ACL-4	CRL-5878
NCI-H1650	Adenocarcinoma	x	V225_splice	ELREA746del	RPMI	CRL-5883
NCI-H1651	Adenocarcinoma	x	C176Y	x	ACL-4	CRL-5884
NCI-H1693	Adenocarcinoma	x	Q331_splice	x	RPMI	CRL-5887
NCI-H1703	Adenocarcinoma	x	A307_splice	x	RPMI	CRL-5889
NCI-H1792	Adenocarcinoma	G12C	E224_splice	x	RPMI	CRL-5895
NCI-H1793	Adenocarcinoma	x	R209*	C311F	HITES	CRL-5896
NCI-H1944	Adenocarcinoma	G13D	x	x	RPMI	CRL-5907
NCI-H1975	Adenocarcinoma	x	x	T790M, L858R	RPMI	CRL-5908
NCI-H2009	Adenocarcinoma	G12A	R273L	Intron	HITES	CRL-5911
NCI-H2030	Adenocarcinoma	G12C	G262V	x	RPMI	CRL-5914
NCI-H2085	Adenocarcinoma	x	ND	ND	ACL-4	CRL-5921
NCI-H2122	Adenocarcinoma	G12C	C176F, Q16L	x	RPMI	CRL-5985
NCI-H2126	Adenocarcinoma	x	E62*	x	ACL-4	CCL-256
NCI-H23	Adenocarcinoma	G12C	M246I	x	RPMI	CRL-5800
NCI-H2405	Adenocarcinoma	x	x	x	ACL-4	CRL-5944
NCI-H322	Adenocarcinoma	x	R248L	x	RPMI	CRL-5806
NCI-H358	Adenocarcinoma	G12C	x	x	RPMI	CRL-5807
NCI-H441	Adenocarcinoma	G12V	R158L	x	RPMI	HTB-174

Lung Cancer Cell Line	Subtype	K-Ras Mut	P53 Mut	EGFR Mut	Media	ATCC Number
NCI-H460	Large cell	Q61H	x	x	RPMI	HTB-177
NCI-H520	Squamous	x	W146*	x	RPMI	HTB-182
NCI-H522	Adenocarcinoma	x	P191fs FRAME SHIFT DEL	x	RPMI	CRL-5810
NCI-H661	Large cell	x	R158L, S215I	x	RPMI	HTB-183
NCI-H838	Adenocarcinoma	x	x	x	RPMI	CRL-5844
HCC4006	Adenocarcinoma	x	x	ELR746del INFRAME	RPMI	CRL-2871
SK-LU-1	Adenocarcinoma	G12D	H193R	x	EMEM	HTB-57
SK-MES-1	Squamous	x	E298*	x	EMEM	HTB-58
SW 1573	Squamous	G12C	Intron	x	RPMI	CRL-2170

Supplementary Table 2

**Breast Cancer Cell Line**	**Screen 1**	**Screen 2**	**Screen 3**	**Screen 4**
AU565		x	x	x
BT-20		x	x	x
BT-474	x	x	x	x
BT-483		x	x	x
BT-549	x	x	x	
CAMA-1		x	x	x
HCC1143		x	x	x
HCC1395		x	x	x
HCC1419		x	x	x
HCC1500		x		
HCC1569		x	x	x
HCC1599		x	x	
HCC1806		x	x	x
HCC1937		x	x	x
HCC1954		x	x	x
HCC2218		x	x	x
HCC38		x	x	x
HCC70		x	x	x
Hs 578T	x	x	x	x
JIMT-1		x	x	x
MCF7	x	x	x	x
MDA-MB-134-VI		x	x	x
MDA-MB-157		x	x	x
MDA-MB-175-VII		x	x	x
MDA-MB-231	x	x	x	x
MDA-MB-415		x	x	x
MDA-MB-436		x	x	x
MDA-MB-453		x	x	x
MDA-MB-468		x	x	x
MDA-MB-361	x	x	x	
SK-BR-3		x	x	x
T-47D	x	x	x	x
UACC-812		x	x	x
ZR-75-1		x	x	x
ZR-75-30		x		

Lung Cancer Cell Line	Screen 1	Screen 2
A549		x
Calu-3		x
NCI-H1155		x
NCI-H1355		x
NCI-H1373		x
NCI-H1395		x
NCI-H1437		x
NCI-H1563	x	x
NCI-H1581	x	x
NCI-H1650	x	x
NCI-H1651		x
NCI-H1693		x
NCI-H1703		x
NCI-H1792		x
NCI-H1793		x
NCI-H1944	x	x
NCI-H1975	x	x
NCI-H2009		x
NCI-H2030		x
NCI-H2085		x
NCI-H2122		x
NCI-H2126		x
NCI-H23	x	x
NCI-H2405		x
NCI-H322		x
NCI-H358		x
NCI-H441		x
NCI-H460		x
NCI-H520	x	x
NCI-H522		x
NCI-H661	x	x
NCI-H838		x
HCC4006	x	x
SK-LU-1		x
SK-MES-1		x
SW 1573		x

Supplementary Table 3

	Halistanol sulfate (mkh2-141-2)		Topsentinol L trisulfate (mkh2-141-4)	
Position	δ_C (mult.)	δ_H (mult., *J* in Hz)	δ_C (mult.)	δ_H (mult., *J* in Hz)
1	39.4	2.07 (br d, 14.3)	39.4	2.07 (m)
		1.48 (dd, 15.4, 2.9)		1.48 (dd, 14.9, 3.7)
2	75.7	4.80 (s)	75.7	4.80 (s)
3	75.6	4.75 (s)	75.6	4.75 (br d, 22.3)
4	25.2	2.28 (br d, 14.6)	25.2	2.28 (br d, 14.5)
		1.79 (br t, 13.4)		1.78 (m)
5	45.5	1.63 (m)	45.5	1.62 (m)
6	78.9	4.19 (td, 11.1, 4.4)	78.9	4.19 (td, 11.1, 4.4)
7	40.2	2.37 (dt, 12.2, 4.3)	40.2	2.36 (dt, 12.1, 4.3)
		1.05 (m)		1.04 (m)
8	35.3	1.53 (m)	35.3	1.53 (m)
9	56.0	0.76 (m)	56.0	0.75 (m)
10	37.8		37.8	
11	22.0	1.54 (m)	22.0	1.55 (m)
		1.31 (m)		1.31 (m)
12	41.3	2.00 (br d, 12.4)	41.2	2.00 (dt, 12.4, 2.9)
		1.15 (m)		1.17 (m)
13	43.9		43.8	
14	57.8	1.11 (m)	57.8	1.11 (m)
15	25.3	1.63 (m)	25.3	1.59 (m)
		1.11 (m)		1.11 (m)
16	29.3	1.86 (m)	30.3	1.73 (m)
		1.29 (m)		1.29 (m)
17	57.5	1.16 (m)	57.5	1.19 (m)
18	12.6	0.69 (s)	12.8	0.71 (s)
19	15.4	1.05 (s)	15.4	1.05 (s)
20	37.8	1.38 (m)	42.2	2.06 (m)
21	19.7	0.94 (d)	21.9	1.05 (d, 7.2)
22	36.8	1.56 (m)	140.8	5.14 (dd, 15.2, 8.5)
		0.90 (m)		NA
23	29.4	1.63 (m)	128.5	5.05 (dd, 15.6, 9.5)
		0.71 (m)		NA
24	45.5	0.99 (m)	57.8	1.34 (m)
25	34.2		29.9 [a]	1.70 (m) [a]
26	27.9	0.86 (s)	22.3 [a]	0.86 (dd, 6.6, 1.6) [a]
27	27.9	0.86 (s)	19.8 [a]	0.80 (dd, 6.7, 4.7) [a]
28	15.1	0.83 (d)	29.9 [b]	1.70 (m) [b]
29	27.9	0.86 (s)	22.2 [b]	0.86 (dd, 6.6, 1.6) [b]
30	NA	NA	19.6 [b]	0.80 (dd, 6.7, 4.7) [b]

[a] Chemical shifts cannot be distinguished from those denoted by [b].

Supplementary Table 4

Protein	Paired TTEST	DMSO Av	TLT Av	Mean diff DMSO-M4	% Change
14-3-3-beta	0.297467434	0.1205898	0.17484	-0.054254273	31.03008862
14-3-3-epsilon	0.127055041	0.0924194	0.08764	0.004775493	-5.448744719
14-3-3-zeta	0.611359948	0.6997833	0.69274	0.007044538	-1.016911279
4E-BP1	0.88102686	0.2872652	0.286	0.001265921	-0.442630957
4E-BP1_pS65	0.688000793	0.6132918	0.60668	0.006608546	-1.089290952
4E-BP1_pT37_T46	0.052849589	0.7249001	0.83875	-0.11385355	13.57413446
53BP1	0.129295665	0.4471605	0.50738	-0.060216947	11.86827429
ACC_pS79	0.023981823	0.6414503	0.51904	0.122410764	-23.5840916
ACC1	0.057831968	0.7090081	0.66169	0.047320333	-7.151459425
ACVRL1	0.204285214	0.0932188	0.09812	-0.004902111	4.995992268
ADAR1	0.722298854	0.1220255	0.12328	-0.001252066	1.015647984
Akt	0.019548209	0.572984	0.52345	0.04953045	-9.462243974
Akt_pS473	0.956418547	0.3849057	0.38686	-0.001955655	0.505518217
Akt_pT308	0.848831423	1.0898163	1.07076	0.019051852	-1.779275781
AMPK-alpha	0.494529968	0.8115008	0.82971	-0.01821201	2.194977551
AMPK-alpha_pT172	0.030926158	0.2468539	0.18237	0.06448569	-35.360163
Annexin-I	0.202592026	0.2944506	0.35136	-0.056913782	16.19793713
Annexin-VII	0.479745507	0.1808896	0.19652	-0.015631946	7.954316733
AR	0.086105524	0.1613788	0.15309	0.008291424	-5.416139865
A-Raf	0.021802658	0.3242939	0.30103	0.023259764	-7.726620108
ARHI	0.374227021	0.0561487	0.0545	0.001644846	-3.017852599
ATM	0.310104666	0.1761461	0.17153	0.004614255	-2.690027967
ATM_pS1981	0.937022455	0.0777696	0.07816	-0.000390239	0.499282649
ATP5H	0.186610373	0.0743909	0.07776	-0.003372651	4.337059112
ATR	0.92837797	0.2607643	0.26007	0.000696483	-0.26780838
Bad_pS112	0.010420165	0.4945067	0.55515	-0.060640319	10.9232895
Bak	0.008721632	0.1909752	0.2054	-0.0144239	7.022377178
BAP1	0.259977565	0.2223549	0.21511	0.007244081	-3.367604315
Bax	0.343854359	0.3507281	0.36731	-0.016583733	4.514891461
b-Catenin	0.584632507	0.6792402	0.71601	-0.036766439	5.134930087
b-Catenin_pT41_S45	0.070481607	0.1303322	0.13996	-0.009625835	6.877656406
Bcl2	0.422689711	0.0737728	0.07546	-0.001686947	2.235559768
Bcl-xL	0.13933645	0.5464368	0.57938	-0.032944392	5.68613386
Beclin	0.302512426	0.1887283	0.22117	-0.032440994	14.66794427
Bid	0.654051893	0.2214305	0.22455	-0.003117031	1.388138801
Bim	0.601368016	0.5163407	0.53035	-0.014011711	2.641962346
B-Raf	0.032395628	1.009608	0.94963	0.059981572	-6.316333569
B-Raf_pS445	0.778696211	0.1716865	0.17297	-0.001285076	0.742940326
BRCA2	0.634796435	0.1778776	0.18588	-0.008002506	4.305198123
Caspase-7-cleaved	0.199719195	0.0678278	0.07255	-0.004718936	6.504683391
Caspase-8	0.111844814	0.3088891	0.29177	0.017117839	-5.866869961
Caveolin-1	0.330102248	0.454963	0.44035	0.014615083	-3.31898519
CD29	0.347436034	0.031597	0.03009	0.001504719	-5.00034688
CD31	0.027495297	0.0240145	0.02141	0.002608377	-12.18521243

Protein	Paired TTEST	DMSO Av	TLT Av	Mean diff DMSO-M4	% Change
CD49b	0.000681184	0.090206	0.08321	0.006999799	-8.412591597
CDK1	0.527951409	0.2966172	0.30303	-0.006412135	2.116011117
Chk1	0.517023385	0.4436631	0.43806	0.005598172	-1.277931995
Chk1_pS345	0.012431443	0.2876721	0.24789	0.039780631	-16.0476008
Chk2	0.900777361	0.3054057	0.30321	0.002196345	-0.724366013
Chk2_pT68	0.234845145	0.1516815	0.15712	-0.005434188	3.458717341
c-Jun_pS73	0.039255625	0.2882244	0.34707	-0.058847805	16.95549391
c-Kit	0.137841447	0.1486842	0.15822	-0.009531557	6.024403154
Claudin-7	0.838258667	0.5496062	0.53524	0.014368512	-2.684510622
c-Met	0.100343269	0.149599	0.14234	0.007259813	-5.100362748
c-Met_pY1234_Y1235	0.607157419	0.1791378	0.1818	-0.002660777	1.463584969
c-Myc	0.823539509	0.3123089	0.3083	0.00400808	-1.300054814
Collagen-VI	0.581827632	0.1085949	0.09859	0.010000297	-10.14284249
Complex-II-Subunit	0.019357592	0.4883395	0.5321	-0.043759206	8.223889209
Cox2	0.267016058	0.1049197	0.17838	-0.073460635	41.18203035
Cox-IV	0.21180139	0.0470478	0.04401	0.003040435	-6.908917587
C-Raf	0.00569841	0.2922268	0.26106	0.031170474	-11.94013341
C-Raf_pS338	0.181233222	0.4905809	0.45703	0.033551782	-7.341279215
Cyclin-B1	0.047649153	1.5024802	1.70111	-0.19862495	11.67622972
Cyclin-D1	0.255149632	0.3947857	0.37585	0.018939256	-5.039094233
Cyclin-E1	0.471229306	0.3046325	0.2948	0.009828166	-3.333793248
Cyclophilin-F	0.588089846	1.2412809	1.12366	0.117620772	-10.4676463
DJ1	0.057596359	0.3065183	0.31909	-0.012574353	3.940658557
Dvl3	0.305796393	0.3631395	0.35437	0.008770662	-2.475009209
E2F1	0.465992121	0.0490497	0.05045	-0.001399862	2.774776277
E-Cadherin	0.414543231	0.2309039	0.26116	-0.030252555	11.58407426
eEF2	0.024329272	0.4482607	0.42437	0.023888806	-5.629214761
eEF2K	0.019692069	0.5732332	0.5177	0.055530627	-10.72635653
EGFR	0.694306782	0.6800515	0.66891	0.011142733	-1.665807521
EGFR_pY1068	0.28897535	0.0780788	0.09203	-0.013946575	15.15514849
EGFR_pY1173	0.30499704	0.1638452	0.17395	-0.010102281	5.80766297
eIF4E	0.125804471	0.8195001	0.79346	0.026037641	-3.28152156
eIF4G	0.878724419	1.035565	1.03183	0.003731644	-0.361651783
ER-alpha	0.504178772	0.0388962	0.0377	0.001192711	-3.163397879
ER-alpha_pS118	0.634911633	0.3953871	0.39946	-0.004070958	1.019120408
ERCC1	0.007891293	0.1646311	0.19196	-0.02732517	14.23510243
Ets-1	0.436109849	0.2073012	0.20972	-0.002419888	1.153860178
FAK	0.00596371	0.3746999	0.35153	0.023166155	-6.590023246
FAK_pY397	0.080659613	0.0997996	0.08071	0.019094485	-23.65957935
FASN	0.010279931	0.7383777	0.68099	0.057383377	-8.426410979
Fibronectin	0.287511878	0.0348751	0.03279	0.002080442	-6.34384711
FoxM1	0.021099513	0.466201	0.42671	0.039490719	-9.254691723
FoxO3a	0.551797236	0.1353121	0.1374	-0.002085824	1.518089719
FoxO3a_pS318_S321	0.943894769	0.5589604	0.56027	-0.001312249	0.234216082
G6PD	0.228099421	0.0796185	0.07617	0.003448534	-4.52742041

Protein	Paired TTEST	DMSO Av	TLT Av	Mean diff DMSO-M4	% Change
Gab2	0.129420955	0.2553089	0.24282	0.012488801	-5.143232028
GAPDH	0.076587901	0.511228	0.41902	0.092207104	-22.00536928
GATA3	0.444543139	0.2890905	0.27977	0.009317527	-3.330389022
GCN5L2	0.581933997	0.4024414	0.41069	-0.008251661	2.009204067
GPBB	0.693848285	0.2242394	0.23134	-0.00710235	3.070067796
GSK-3ab	0.107780308	0.8094326	0.82645	-0.017016942	2.059041866
GSK-3ab_pS21_S9	0.550735827	0.6241206	0.64794	-0.023816819	3.675790205
GSK-3b_pS9	0.177310106	0.4761323	0.5083	-0.03216499	6.327988207
Gys	0.995834943	0.518761	0.51881	-5.10903E-05	0.009847553
Gys_pS641	0.541992189	0.3732588	0.36622	0.007043452	-1.923308627
HER2	0.263192338	0.0281805	0.02605	0.002128034	-8.16827614
HER2_pY1248	0.104377998	0.0403153	0.04791	-0.0075911	15.8456832
HER3	0.192076665	0.2256087	0.20685	0.018756371	-9.06751822
HER3_pY1289	0.334961404	0.3471657	0.33777	0.009395071	-2.781494189
Heregulin	0.356306262	0.158463	0.16414	-0.005672826	3.456177062
HIAP	0.418473854	0.4569881	0.44928	0.00770568	-1.715108173
Histone-H3	0.859920263	0.1358833	0.13816	-0.002276057	1.647415041
IGF1R-beta	0.633604069	0.2032754	0.20619	-0.002917845	1.415102345
IGFBP2	0.004643877	0.1284908	0.19396	-0.065470005	33.7542394
INPP4b	0.224786181	0.1661943	0.16219	0.004006157	-2.470067174
IRS1	0.149211343	0.6053276	0.58946	0.015868518	-2.692047293
JAB1	0.551441944	0.0690752	0.06787	0.001201038	-1.769508052
JNK_pT183_Y185	0.622517253	0.1801214	0.18676	-0.006641022	3.555867298
JNK2	0.620583516	0.4413883	0.43619	0.005199641	-1.192062542
Lck	0.404041823	0.1615087	0.15269	0.008820444	-5.776766198
MAPK_pT202_Y204	0.063897497	0.3657461	0.27199	0.09375541	-34.47007848
Mcl-1	0.869330062	0.3021391	0.30475	-0.002608929	0.856093758
MDM2_pS166	0.184798884	0.4228332	0.43978	-0.016949566	3.854077221
MEK1	0.749093243	0.4931058	0.48759	0.005517333	-1.131555193
MEK1_pS217_S221	0.330871457	0.4264982	0.40648	0.020019075	-4.924994372
MEK2	0.046725637	0.262228	0.24935	0.012881695	-5.166185233
Merlin	0.110425164	0.6224777	0.57406	0.048420054	-8.434701674
MIG6	0.65818707	0.2714421	0.26646	0.004981295	-1.869428612
MSH2	0.232864132	0.7096262	0.69032	0.019305584	-2.796611216
MSH6	0.353124034	0.8008488	0.8208	-0.019953461	2.43097042
mTOR	0.10247673	1.0000496	0.97459	0.025457724	-2.612141883
mTOR_pS2448	0.535634771	0.5736505	0.58052	-0.006872585	1.183860668
Myosin-11	0.130662479	0.2516351	0.24104	0.010593952	-4.3950794
Myosin-IIa_pS1943	0.136330833	0.5590272	0.52052	0.038505586	-7.397499919
NAPSIN-A	0.000244622	0.1888714	0.1789	0.009967334	-5.571328945
N-Cadherin	0.115979527	0.108239	0.0995	0.008736554	-8.780236006
NDRG1_pT346	0.168014205	0.3168807	0.37585	-0.05897111	15.68998963
NF-kB-p65_pS536	0.700093069	0.5674735	0.54107	0.026404604	-4.880082075
Notch1	0.661226615	0.6396916	0.65434	-0.01464397	2.237990814
N-Ras	0.424040717	0.0373128	0.03559	0.001720188	-4.83299624

Protein	Paired TTEST	DMSO Av	TLT Av	Mean diff DMSO-M4	% Change
p16INK4a	0.089057378	0.2589195	0.24097	0.017949144	-7.448692453
p21	0.072152138	0.589791	0.68726	-0.09746523	14.18179013
p27_pT157	0.628732156	0.4132618	0.42135	-0.008085064	1.918861914
p27_pT198	0.015523921	0.380493	0.4207	-0.040209916	9.557793229
p27-Kip-1	0.101448071	0.1353413	0.12438	0.010959469	-8.811151583
p38	0.03267437	0.8570567	0.8278	0.029257917	-3.534423672
p38_pT180_Y182	0.002755169	0.4029123	0.52386	-0.120951629	23.08836548
p38-alpha	0.00774209	0.3541716	0.32024	0.033932183	-10.59587949
p53	0.314234566	0.0975719	0.09485	0.002724253	-2.872240696
p70-S6K_pT389	0.031044202	0.1669332	0.14084	0.026097196	-18.53020434
p70-S6K1	0.172006967	0.2027094	0.1965	0.006210831	-3.160751165
PAI-1	0.360871698	0.7728393	0.79675	-0.02391276	3.001280111
PARP1	0.967407249	2.6880892	2.69449	-0.006399885	0.237517567
PARP-cleaved	0.118830095	0.0705842	0.06733	0.00325635	-4.836555963
Paxillin	0.906536745	0.5151816	0.51694	-0.001762799	0.341003507
PCNA	0.06253059	0.2458135	0.23039	0.015419488	-6.692659587
Pdcd-1L1	0.112960284	0.1537748	0.21549	-0.061714605	28.63927851
Pdcd4	0.2306148	0.4398906	0.47403	-0.034134929	7.201073805
PDGFR-beta	0.638238272	0.7599954	0.79413	-0.034136002	4.298533004
PDK1	0.004328426	0.1989206	0.17397	0.024949699	-14.34130609
PDK1_pS241	0.010023724	0.4803055	0.44315	0.037151425	-8.38341026
PEA-15	0.575925151	0.4603699	0.4734	-0.013030284	2.752488295
PEA-15_pS116	0.07112505	0.4775941	0.51308	-0.035485327	6.91614747
PI3K-p110-alpha	0.045315699	0.3424673	0.32277	0.019699858	-6.103421879
PI3K-p85	0.209222646	0.2749676	0.27946	-0.004493387	1.607876193
PKC-alpha	0.896269303	0.7304674	0.7277	0.002768541	-0.380451526
PKC-alpha_pS657	0.795719345	0.4284552	0.43106	-0.002602363	0.603716011
PKC-beta-II_pS660	0.425784911	0.7686049	0.80879	-0.040182907	4.968287878
PKC-delta_pS664	0.045976568	0.3448394	0.36918	-0.024340036	6.593008843
PMS2	0.025956644	0.801951	0.74703	0.054920915	-7.351901528
Porin	0.243883965	0.0857229	0.08904	-0.003322	3.730702144
PR	0.663238212	0.1169763	0.11619	0.000789801	-0.679769967
PRAS40_pT246	0.365275156	0.8290985	0.86475	-0.035650351	4.122624665
PREX1	0.101304994	0.1049792	0.09812	0.006861336	-6.992955773
PTEN	0.782392774	0.1851321	0.18314	0.001988066	-1.085520762
Rab11	0.858096277	0.0879305	0.08734	0.000591986	-0.677806514
Rab25	0.332786784	0.1389058	0.13712	0.001783779	-1.300869619
Rad50	0.960424116	0.4113952	0.41097	0.000428285	-0.104213934
Rad51	0.024369829	0.1550382	0.13781	0.017233102	-12.50541433
Raptor	0.79377184	0.762859	0.75543	0.007433942	-0.984074094
Rb	0.533730146	0.2241153	0.2198	0.004313551	-1.962473631
Rb_pS807_S811	0.457540895	1.1841248	1.11809	0.066029881	-5.90557026
RBM15	0.957138438	0.4360402	0.43545	0.000592611	-0.13609247
Rictor	0.689051732	0.6479663	0.6408	0.007167023	-1.11845059
Rictor_pT1135	0.017269911	0.6853876	0.62371	0.061679537	-9.889167527

Protein	Paired TTEST	DMSO Av	TLT Av	Mean diff DMSO-M4	% Change
RSK	0.147394136	0.4682432	0.45481	0.013431948	-2.953301246
S6_pS235_S236	0.09647995	1.2531184	1.40306	-0.149946424	10.68706343
S6_pS240_S244	0.118730496	0.8496313	0.94022	-0.090591207	9.635081836
SCD	0.759737499	0.0673423	0.06663	0.000707615	-1.061931555
SETD2	0.041455038	0.1748269	0.16728	0.007542015	-4.508486479
SF2	0.082190928	0.0562108	0.05337	0.002836706	-5.314761793
Shc_pY317	0.413441153	0.3421638	0.33226	0.009902927	-2.980467043
Smac	0.20574698	0.4343177	0.36964	0.064677673	-17.4974742
Smad1	0.109784621	0.2190075	0.20699	0.012015241	-5.804681259
Smad3	0.042150885	0.5352521	0.5145	0.020756397	-4.034318757
Smad4	0.109781972	0.1975331	0.18686	0.01066866	-5.709304748
Snail	0.021853524	0.2435867	0.32022	-0.076637945	23.93255874
Src	0.921787018	0.2637837	0.26281	0.000976124	-0.371421462
Src_pY416	0.208694232	0.1672131	0.15217	0.015041977	-9.884909758
Src_pY527	0.10778136	0.700054	0.642	0.05805016	-9.042026273
Stat3_pY705	0.061361639	0.288247	0.23968	0.048567263	-20.26340212
Stat5a	0.004748425	0.5811547	0.52104	0.060117804	-11.53810864
Stathmin-1	0.452980835	0.1374042	0.14212	-0.004714313	3.317171496
Syk	0.804047646	0.2295709	0.22859	0.000979491	-0.428489726
TAZ	0.293525898	0.4116032	0.58673	-0.175121832	29.8473411
TFRC	0.45006093	0.5982809	0.62051	-0.022225227	3.581790061
TIGAR	0.203513955	0.4447832	0.43667	0.008111844	-1.857654354
Transglutaminase	0.347628428	0.3099899	0.31823	-0.008237679	2.588612494
TSC1	0.724792685	0.6995556	0.70621	-0.006652592	0.942015755
TTF1	0.072765875	0.4216622	0.40529	0.016367572	-4.038438288
Tuberin	0.007348867	1.2912628	1.17141	0.119849468	-10.23118546
Tuberin_pT1462	0.472275507	0.3208728	0.30659	0.014281069	-4.658009245
TWIST	0.026724298	0.0787599	0.07013	0.00863235	-12.30950185
Tyro3	0.148506776	0.2903453	0.26374	0.026606514	-10.08820479
UBAC1	0.084205337	0.4482809	0.42428	0.023999931	-5.656613047
UGT1A	0.129590932	0.0798728	0.07732	0.002550457	-3.298473152
UQCRC2	0.968988613	0.097701	0.09758	0.000124445	-0.127535891
VEGFR-2	0.399973553	0.4634747	0.45786	0.005619443	-1.227340541
XRCC1	0.599402963	0.4539372	0.45771	-0.003775075	0.824770285
YAP	0.389819715	0.3792817	0.38599	-0.006706443	1.737473896
YAP_pS127	0.786380304	0.7320844	0.73928	-0.007192785	0.972948256
YB1	0.055196453	0.6676773	0.7363	-0.068626495	9.320405293
YB1_pS102	0.019015082	0.6649866	0.78032	-0.11532871	14.77975748

Supplementary Table 5

	Topsentinol L Trisulfate EC50 (μM)			
**Cell Line**	**GFP**	**AMPK**	**CHK1**	**AMPK+CHK1**
**HCC1143**	147.37	73.35	46.60	82.49
**MDA-MB-436**	88.80	81.74	61.86	57.34
